# Dogs as a Natural Animal Model of Epilepsy

**DOI:** 10.3389/fvets.2022.928009

**Published:** 2022-06-22

**Authors:** Wolfgang Löscher

**Affiliations:** ^1^Department of Pharmacology, Toxicology and Pharmacy, University of Veterinary Medicine, Hannover, Germany; ^2^Center for Systems Neuroscience, Hannover, Germany

**Keywords:** seizures, antiseizure medications, pharmacokinetics, intracranial EEG, responsive neurostimulation, status epilepticus, canine epilepsy

## Abstract

Epilepsy is a common neurological disease in both humans and domestic dogs, making dogs an ideal translational model of epilepsy. In both species, epilepsy is a complex brain disease characterized by an enduring predisposition to generate spontaneous recurrent epileptic seizures. Furthermore, as in humans, status epilepticus is one of the more common neurological emergencies in dogs with epilepsy. In both species, epilepsy is not a single disease but a group of disorders characterized by a broad array of clinical signs, age of onset, and underlying causes. Brain imaging suggests that the limbic system, including the hippocampus and cingulate gyrus, is often affected in canine epilepsy, which could explain the high incidence of comorbid behavioral problems such as anxiety and cognitive alterations. Resistance to antiseizure medications is a significant problem in both canine and human epilepsy, so dogs can be used to study mechanisms of drug resistance and develop novel therapeutic strategies to benefit both species. Importantly, dogs are large enough to accommodate intracranial EEG and responsive neurostimulation devices designed for humans. Studies in epileptic dogs with such devices have reported ictal and interictal events that are remarkably similar to those occurring in human epilepsy. Continuous (24/7) EEG recordings in a select group of epileptic dogs for >1 year have provided a rich dataset of unprecedented length for studying seizure periodicities and developing new methods for seizure forecasting. The data presented in this review substantiate that canine epilepsy is an excellent translational model for several facets of epilepsy research. Furthermore, several techniques of inducing seizures in laboratory dogs are discussed as related to therapeutic advances. Importantly, the development of vagus nerve stimulation as a novel therapy for drug-resistant epilepsy in people was based on a series of studies in dogs with induced seizures. Dogs with naturally occurring or induced seizures provide excellent large-animal models to bridge the translational gap between rodents and humans in the development of novel therapies. Furthermore, because the dog is not only a preclinical species for human medicine but also a potential patient and pet, research on this species serves both veterinary and human medicine.

## Introduction

Domestic dogs (*Canis lupus familiaris*) provide an ideal model for translational medicine as they have the most phenotypic diversity and known naturally occurring diseases of all land mammals other than humans (1). Dogs share an evolutionary history and high amount of ancestral genetic sequence with humans, as well as the characteristics of our environment (2). The level of sophistication of the healthcare system for dogs in Europe and the United States is second only to that of humans. Thus, data related to dog health presents many opportunities to discover insights into health and disease outcomes in both dog and human populations. In fact, naturally occurring diseases in companion animals are often similar—and sometimes identical—to human diseases concerning the disease etiology, progression, and how that disease responds to medical intervention or treatment (1, 3, 4). In addition, dogs are the main non-rodent species in preclinical drug development, particularly in the evaluation of pharmaceutical safety, pharmacokinetics, and efficacy (5–7). Concerning translational neuroscience, it is important to note that unlike the lissencephalic brains of mice and rats, the brains of both dogs and humans are gyrencephalic (2).

Epilepsy is the most common medical neurologic disease of dogs (8). While reference to using dogs with naturally occurring epilepsy as a potential comparative model of the underlying basis and therapy of epilepsy was made in the 1970's (9, 10), we were the first to perform comparative pharmacokinetic studies on anti-seizure medications (ASMs; previously termed antiepileptic drugs) in dogs (11–24). We proposed epileptic dogs as a natural model of human epilepsy in research and drug development some 40 years ago (24–26) followed by numerous studies in this species, including the first controlled clinical drug trial in epileptic dogs (27). This review will highlight the usefulness of dogs with naturally occurring or induced seizures as a large animal model of epilepsy with a focus on pharmacology and drug development. In this respect, it is important to note that research on this species serves both veterinary and human medicine, as the epileptic dog is not only a preclinical species for advancing knowledge and treatment for humans, but also a potential patient as a pet. To emphasize the biomedical and societal importance of this aspect, we will use the development of the ASM imepitoin for canine epilepsy as an example.

## Epilepsy in Dogs

### Epidemiology of Epilepsy in Dogs

In both dogs and humans, epilepsy is a complex brain disease characterized by an enduring predisposition to generate recurrent epileptic seizures. Epilepsy is not a single disease but a group of disorders characterized by a broad array of clinical signs, age of onset, and underlying causes (28). The true prevalence of epilepsy in dogs is unknown and has been estimated to be 0.6–0.75% in the general dog population (29, 30), which is similar to the prevalence of epilepsy in humans (28). In certain dog breeds predisposed to idiopathic epilepsy, considerable higher prevalence rates are reported than those estimated for the general dog population, which is one of the reasons a genetic component is suspected in certain canine breeds (31).

The International Veterinary Epilepsy Task Force (IVETF) divides epilepsy into the categories of structural epilepsy (due to acquired or inherited structural brain alterations) and idiopathic epilepsy (32). Idiopathic epilepsy is defined as a disease in its own right where no structural cerebral pathology is suspected (or seen) and in many cases, a genetic component may be involved (32). In this respect, the terminology of the IVETF differs from the terminology of the International League Against Epilepsy (ILAE) for human epilepsy, in which “idiopathic” has been replaced by “genetic” and “unknown etiology” (33). Based on seizure types, epilepsies are classified into focal, generalized, generalized and focal, and unknown (33). At the next level, the ILAE differentiates numerous epilepsy syndromes by a distinctive clinical pattern and electroencephalographic (EEG) features (34), which is not possible yet in canine epilepsy because of the limitations of EEG analyses in dogs (see below). Well-recognized examples of epilepsy syndromes in humans include childhood absence epilepsy, juvenile myoclonic epilepsy (JME), and benign epilepsy with centrotemporal spikes (33). A final level of diagnosis of the ILAE classification scheme establishes that the primary etiology and epilepsy diagnosis have been determined (33). This level of diagnosis opens the gateway to a precision-medicine approach that reflects current scientific efforts (35). In medicine, the ability to make an etiological diagnosis is rapidly increasing with the revolution in genetics and other fields such as neuroimaging. Numerous new etiological diagnoses are emerging, particularly pediatric epileptic encephalopathies (36, 37). One of the best-known examples is the Dravet syndrome, which is caused by a known mutation of the sodium channel gene SCN1A (35). Because of the limited availability of EEG-video, genetic, and brain imaging data, canine classification of epilepsy is mainly based on presumed etiology.

**Figure 1:**
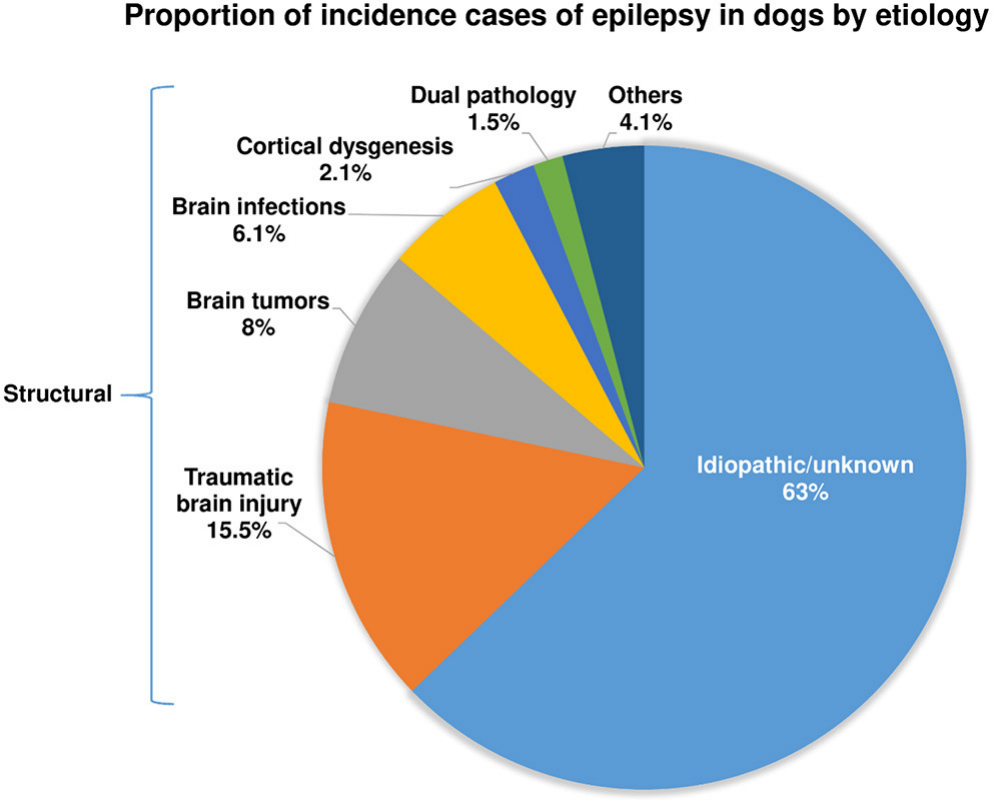
Presumed causes of recurrent epileptic seizures in dogs with epilepsy. See Steinmetz et al. (38) and text for further details.

In 2013, we published the outcome of a large retrospective study in 1,000 dogs referred to the Department of Small Animal Medicine and Surgery of our University in Hannover over 11.5 years (38). As shown in Figure 1, 63% of the dogs were categorized as having idiopathic or unknown etiology, and 37% had a structural etiology. Within the group of structural or acquired epilepsy, dogs with traumatic brain injury (TBI) formed the largest subgroup. More recently, similar data were reported by Hall et al. (39). Based on a retrospective study on 900 dogs undergoing magnetic resonance imaging (MRI) for seizures, structural lesions were identified as a cause of seizures in 45.1% of cases, and no structural lesions were identified in 54.9% of cases. In the structural epilepsy group, TBI was less often identified as a cause of acquired epilepsy than in our study, which may be due to the differences in study design or case population (39). A similar figure of 46% of epileptic dogs having structural causes was obtained in a prospective study by Podell et al. (40).

Importantly, the epidemiologic data on predisposing causes of epilepsy in dogs shown in Figure 1 (38) are very similar to respective studies in humans with epilepsy (41–43). Based on the relative proportion of etiologies identified in a large population-based study out of Rochester, Minnesota, U.S.A., over 50 years (44), 65% of the patients were categorized as “idiopathic/cryptogenic” and 35% as symptomatic. In the latter group, head trauma was identified as the cause of epilepsy in 6% of the population, stroke in 10%, brain tumors in 6%, infections in 3%, degenerative causes in 4%, and congenital brain alterations in 8%, respectively. This remarkable similarity with the predisposing causes of epilepsy in dogs shown in Figure 1 is a strong argument for the suitability of epileptic dogs as a translational model of epilepsy. However, it is important to note that in human medicine the percentage of “cryptogenic” epilepsies (now termed epilepsies with unknown etiologies) is progressively declining in recent years because of the frequent use of high-resolution MRI and the advent of modern technologies to identify genetic causes, such as next-generation sequencing (42, 45, 46). It is to be expected that similar advances will take place concerning epilepsy in dogs in the future. The recent MRI-based study of Hall et al. (39) is a good example because the percentage of dogs without obvious structural lesions was only 54.9 percent of cases, which is ~8% lower than in the 2013 study of Steinmetz et al. (38).

Dog breeds, which have been identified as being predisposed to idiopathic epilepsy, include the Australian Shepherd, Belgian Tervueren, Belgian Shepherd, Border Collie, Irish Wolfhound, Labrador Retriever, Petit Basset Griffon Vendeen, Finnish Spitz Dog, and Italian Spinone (31, 47). Even though pedigree analysis has strongly suggested genetic influence in these breeds, the identification of the affected genes has been quite difficult (47–49). Up to date, only a few monogenic epilepsies have been identified in dogs that parallel epilepsies in humans regarding epilepsy onset and seizure types (47). Thus, in contrast to the genetics of inherited human epilepsies, where modern techniques such as high-throughput sequencing have led to the identification of a progressively increasing high number of epilepsy syndromes, including the epileptic encephalopathies, with known genetic basis (36, 42, 45, 50–52), this area of research is in its infancy in canine epilepsy.

### Seizure Types in Dogs With Epilepsy

According to the ILAE, epileptic seizures are divided into focal onset, generalized onset, and unknown onset (53). Generalized onset seizures are subdivided into motor (e.g., generalized tonic-clonic) and non-motor (e.g., absence) seizures. Focal onset seizures may secondarily generalize to generalized tonic-clonic seizures. In principal, these seizure types are also observed in epileptic dogs. In the past, generalized tonic-clonic seizures were often considered the most frequent type of seizures in canine epilepsy, but accumulating evidence suggests that focal onset seizures are the major seizure onset form in canine epilepsy (32, 54, 55). As in humans, generalized tonic-clonic seizures may have a generalized onset or arise by secondary generalization after focal onset seizures. In dogs, seizure type (e.g., focal vs. generalized) should not be used as an isolated variable to predict the presence of structural epilepsy, although focal (partial) seizures often suggest a structural etiology (40, 56). In general, the type of epilepsy and seizures is an important factor for the prognosis of therapy (56). Structural epilepsies with focal onset seizures have a poorer prognosis than idiopathic epilepsies with generalized onset seizures (57). Focal onset seizures may be very subtle and can be easily missed by the dog's owner, particularly when they occur at the night. More complex focal seizures may manifest as bizarre behavior, such as unprovoked aggression, running uncontrollably, or rhythmic barking (32). Furthermore, structural epilepsy with focal onset seizures may be associated with a pre-ictal phase, i.e., is a period of altered behavior in which the dog may hide, appear nervous, or seek out the owner. Although the literature on ictal semiology of focal seizures in dogs is limited, similarities have been found with regard to the distribution and semiology of focal seizures between dogs and humans (58). As in humans (28), focal seizures with or without secondary generalization seem to be the most frequent type of seizures in dogs with epilepsy, associated with a poor prognosis of treatment (55).

### EEG Studies in Dogs With Epilepsy

In a clinical setting, non-invasive scalp EEG recording by a standardized electrode arrangement is a key method in the evaluation of epilepsy in humans, guiding primary diagnosis, epilepsy classification, and treatment (59, 60). In contrast, the EEG has never been established as a routine laboratory test for the diagnosis of canine epilepsy, at least in part because non-invasive scalp EEG recording is compromised by artifacts due to the thick muscles on the dog's skull (61). To overcome this problem, subdermal needle scalp electrodes have been used in specific neurological referral hospitals, but this necessitates immobilization of the dog by deep sedation or anesthesia, which is likely to affect interictal and ictal EEG recordings (62). To reduce this problem, when sedation or general anesthesia was used for EEG electrode placement, ambulatory EEG recording may extend beyond recovery to a normal mentation state (63). The IVETF (32, 56) has recognized and described the importance of the EEG in epileptic dogs and noted that the development of a standardized EEG protocol is an urgent priority for veterinary neurology, not the least to promote resective epilepsy surgery in the future. Indeed, surgical removal of the epileptic focus is the only available cure for epilepsy (64), but is not yet used in dogs with drug-resistant epilepsy (DRE) because it is difficult to accurately localize the origin of seizures in the brain of this species (65, 66).

There are various reports of EEG recordings in epileptic dogs, but most likely due to the sedation or anesthesia used, the detection rates of EEG alterations in most reports were low (66). It is unlikely that EEG recordings in epileptic dogs can be used to characterize seizures unless novel implantable EEG devices and continuous EEG monitoring become available. The group of Brian Litt at the University of Pennsylvania and collaborators have developed a novel implanted device to wirelessly record and analyze continuous intracranial canine EEG (67). When using this device for continuous intracranial EEG (iEEG) monitoring in six conscious (non-anesthetized) dogs with naturally occurring epilepsy over 5 months, Davis et al. (67) demonstrated previously uncharacterized intracranial seizure onset patterns in these animals that are strikingly similar in appearance to human focal onset epilepsy. In a subsequent yearlong study with this device in four epileptic dogs, Ung et al. (68) found significant temporal variability in seizures and interictal bursts after electrode implantation that required several weeks to reach a steady-state. These findings, comparable to those reported in humans implanted with the NeuroPace Responsive Neurostimulator System (RNS) device (see below), suggest that transient network changes following electrode implantation may need to be taken into account when interpreting or analyzing iEEG during evaluation for epilepsy surgery. Once a steady-state was reached, multiple seizure types were observed in each dog, with significant temporal variation between types (68). Seizures typically occurred in clusters, and isolated seizures were rare (see below for a more detailed discussion of this iEEG device).

Morita et al. (69) used continuous EEG recording with subcutaneous electrodes every 1–3 months under sedation with xylazine in epileptic Shetland Sheedogs and found that an epileptic focus was initially detected in the frontal lobe, particularly the internal area, and that paroxysmal foci developed diffusely in other lobes of affected dogs with recurrent convulsions. These examples illustrate the usefulness of continuous EEG recordings in canine epilepsy in localizing the onset of seizures and characterizing their evolution.

### Status Epilepticus in Dogs With Epilepsy

Status epilepticus (SE), the condition of ongoing seizures or repetitive seizure activity without recovery of consciousness between seizures, is one of the more common neurological emergencies with a risk of high mortality or morbidity in people (70). Most frequently, SE is characterized by generalized convulsive tonic-clonic seizures, whereas non-convulsive SE is less frequent. SE may occur in patients with previous epilepsy or acute disorders of the CNS (71). Common causes of SE in human patients with epilepsy include low ASM levels or abrupt termination of treatment with ASMs. SE requires immediate i.v. treatment with an ASM to reduce mortality (72). However, not all patients will respond to initial treatment. The two most important variables that influence the drug response of SE are the underlying etiology and the duration of SE (73). Concerning SE duration, the longer the SE persists (typically ~0.5–1 h), the more likely is the SE to be unresponsive to drug therapy, the higher the mortality, and the worse the long-term consequences are in survivors. Based on treatment response, SE is divided into four stages: early, established, refractory, and super-refractory (74). Initial i.v. treatment with benzodiazepines (BDZs) has become the standard of care for early SE. When treatment fails (“established SE”), a second-line ASM is injected. If this treatment fails, too, SE is defined as refractory, potentially necessitating anesthetic agents to terminate SE (75). Refractory SE occurs in 23–43% of patients with SE and is associated with short-term fatality rates between 16 and 39% (75). Super-refractory SE is defined as seizure activity >24 h despite treatment with anesthetic agents. This includes cases in which seizures recur with an attempted withdrawal of the anesthetics (76). Effective treatment of SE is critical as morbidity and mortality increase dramatically the longer convulsive SE persists.

It has been estimated that nearly 60% of epileptic dogs may—at some point in their lifetime—experience one or more SE events (77). SE may be the first manifestation of a seizure disorder in dogs (78). It results from the failure of endogenous termination of an isolated seizure. The prognosis for dogs with SE is quite poor—up to 25% of affected dogs will not survive hospital discharge (78, 79). SE can lead to permanent brain damage (e.g., neuronal cell necrosis, network reorganization, gliosis) and severe systemic complications (e.g., cardiorespiratory collapse, shock, acidosis, and electrolyte imbalances) (80). Cluster seizures may be a precursor of SE and are defined as two or more seizures within 24 h. However, they differ from SE because, during cluster seizures, patients regain consciousness, or return to baseline CNS function, between seizures (81). As in humans, the main goals of treatment of SE or cluster seizures in dogs are to halt seizure activity, prevent further seizures, identify the cause of the seizures, and manage any complications (79). Effective ASMs in canine SE are the same as are used in humans with SE, making canine SE a translational platform for human therapeutic trials (77).

SE is typically treated by i.v. administration of ASMs in a hospital setting. As in humans, i.v. BDZs are the first-line treatment of SE in dogs. However, when i.v. access is not available for emergency treatment, intramuscular, rectal, intranasal, buccal or sublingual administration may be useful. Charalambous et al. (82) performed a randomized parallel-group clinical trial on intranasal midazolam vs. rectal diazepam for the management of canine SE and found that intranasal midazolam is a quick, safe, and effective first-line medication for controlling SE in dogs and appears superior to rectal diazepam. However, in 30% of the dogs, intranasal midazolam did not terminate the SE. In this respect, it is important to note that the subtypes of SE [early (BDZ-responsive) SE, established SE, refractory SE, super-refractory SE] described above for people have been applied to dogs (80). Furthermore, as in humans, the longer the SE persists before the onset of treatment, the higher the likelihood of drug resistance (83). Combinatorial therapies may be more effective to interrupt SE than single drug treatment (84).

### Sudden Unexpected Death in Epilepsy (SUDEP) in Dogs With Epilepsy

Sudden unexpected death in epilepsy (SUDEP) has been defined in persons with epilepsy as “the sudden, unexpected, witnessed or unwitnessed, non-traumatic, and non-drowning death of a patient with epilepsy with or without evidence of a seizure, excluding documented status epilepticus, and in which postmortem examination does not reveal a structural or toxicological cause of death” (85). SUDEP typically occurs in patients with poorly controlled epilepsy. Although SUDEP is relatively rare, it contributes to the reduced life expectancy of patients with drug-resistant epilepsies (86, 87). Each year, roughly 1 in every 1,000 adults and 1 in 4,500 children with epilepsy will die from SUDEP. The underlying cause of SUDEP is unknown. The condition may be due to an abnormality of breathing, cardiovascular dysfunction, arousal deficits, or a combination of these (87). In dogs, SUDEP is thought to be uncommon but may be underrecognized (78). Probable SUDEP has been documented in a large cohort of dogs with idiopathic epilepsy (88).

### Comorbidities in Dogs With Epilepsy

Comorbidities such as mood and psychiatric disorders or deficits in learning and memory may be present before the onset of epilepsy, may constitute an aspect of the epilepsy syndrome, or occur as a consequence of epilepsy in people (89). Indeed, some common mechanisms, such as structural and functional alterations in the limbic system, might underlie both epilepsy and comorbidities (89). In humans, psychiatric disorders, such as anxiety, depression, psychosis, attention-deficit/hyperactivity disorder (ADHD), and cognitive decline are common comorbidities of epilepsy (28, 90). The prevalence of psychiatric disorders in people with epilepsy is higher than in either the general population or patients with other chronic medical diseases (91).

In epileptic dogs, a variety of comorbid behavioral changes have been reported, including anxiety and defensive aggression, psychosis-like symptoms (e.g., barking without apparent cause, chasing shadows or light spots, aimless pacing and staring into space), ADHD-like symptoms, and cognitive alterations (92, 93). However, abnormal behaviors such as anxiety, restlessness, irritation, and attention-seeking may also constitute prodromal signs that precede the onset of a seizure or post-ictal signs, indicating an involvement of the limbic system. Furthermore, focal seizures with a sensory or psychic component often manifest as behavioral changes, including anxious behaviors, restlessness, pacing, and seeking out their owner (58).

## Treatment of Epilepsy in Dogs

ASMs, previously referred to as anticonvulsant or antiepileptic drugs, are the mainstay of symptomatic epilepsy treatment in humans and dogs (72, 94). The goal of epilepsy therapy is the complete elimination of seizures, which, however, is not always achievable, with a secondary goal to reduce the severity and frequency of seizure events (see below). Currently, about 30 ASMs are available for epilepsy therapy in humans; however, not all are suitable for therapy in dogs. The main reason for this is pharmacokinetic species differences. As shown in Table 1, most ASMs are much more rapidly eliminated in dogs than in humans, making maintenance of therapeutic drug levels in dogs difficult if not impossible. Only three ASMs, phenobarbital, imepitoin, and potassium bromide, have been approved for epilepsy therapy in dogs in Europe, and only one (primidone) in the U.S. Potassium bromide is only approved in Europe as add-on therapy in dogs in which treatment with phenobarbital or imepitoin failed. As in humans, epileptic dogs have to be treated daily and lifelong with an ASM, because the treatment only symptomatically suppresses the seizures. Treatment with too low doses or abrupt termination of treatment may lead to life-threatening SE (see above).

**Table 1:** A comparison of elimination half-lives of antiseizure medications (ASMs) in humans and dogs.

**ASM**	**Half-life (h)**		**Perceived mechanism of action**
	**Human**	**Dog**	
Acetazolamide	10–15	?	Carbonic anhydrase inhibitor
Brivaracetam	7–8	?	SV2A modulator
Cannabidiol (CBD)	24–48	11–19	Unknown (CBD does not act on CB1 or CB2 receptors)
Carbamazepine	25–50^a^,^b^	1–2^a^,^b^	Modulator of voltage-gated sodium channels
Cenobamate	50–60	?	Modulator of voltage-gated sodium channels plus PAM at GABA_A_ receptors
Clobazam	16–50	~1.5	Agonist at the BZD-BS of the GABA_A_ receptor
Clonazepam	18–50	1–3	Agonist at the BZD-BS of the GABA_A_ receptor
Diazepam	24–72^a^ (DMD = 40–130)	1–5^a^ (DMD = 4)	Agonist at the BZD-BS of the GABA_A_ receptor
Eslicarbazepine acetate	10–20	?	Modulator of voltage-gated sodium channels
Ethosuximide	40–60	11–25	Modulator of voltage-gated calcium channels
Felbamate	14–22	4–8	Mixed
Fenfluramine	13–30	2–4	Increase of serotonin release
Fosphenytoin (a prodrug of phenytoin)	~7–15 min^a^ (phenytoin 15–20^b^,^c^)	~3 min^a^ (phenytoin 2–6^b^,^c^)	Modulator of voltage-gated sodium channels
Gabapentin	5–7	3–4	Modulator of α2δ subunit of calcium channels
Imepitoin*	~8	2–6	Partial agonist at the BZD-BS of the GABA_A_ receptor
Lacosamide	13	2–2.5	Modulator of voltage-gated sodium channels
Lamotrigine	21–50	2–5	Modulator of voltage-gated sodium channels
Levetiracetam	6–11	4–5	SV2A modulator
Oxcarbazepine	1–2.5^a^ (MHD = 8–14)	~4^a^ (MHD = 3–4)	Modulator of voltage-gated sodium channels
Perampanel	70	5	Inhibitor of glutamate receptors of the AMPA subtype
Phenobarbital*	70–100^b^	25–90^b^	Partial agonist at the barbiturate-BS of the GABA_A_ receptor
Phenytoin	15–20^b^,^c^	2–6^b^,^c^	Modulator of voltage-gated sodium channels
Potassium bromide*	~300	~600	Potentiation of GABA
Pregabalin	6	6–7	Modulator of α2δ subunit of calcium channels
Primidone**	6–12^a^ (PB = 70–100)	4–12^a^,^b^ (PB = 25–90)	Acts *via* metabolism to PB, which is a partial agonist at the barbiturate-BS of the GABA_A_ receptor
Retigabine (ezogabine)	6–8	3–10	Activator of voltage-gated potassium (K_v_7) channels
Rufinamide	6–10	~10	Mixed
Stiripentol	5–13	?	PAM at GABA_A_ receptors
Sulthiam	2–16	?	Carbonic anhydrase inhibitor
Tiagabin	5–8	1–2	Inhibitor of GAT1 GABA transporter
Topiramate	20–30	3–4	Mixed
Valproate	8–15^a^	1–3^a^	Mixed
Vigabatrin	5–7^d^	?^d^	Inhibitor of GABA degradation
Zonisamide	60–70	~15	Mixed

*Data are from previous reviews of Löscher (95,96) and have been revised and updated for the present review. “?” indicates that no data were found. If not noted otherwise, all half-lives are in hours. Note that dogs eliminate most ASMs much more rapidly than humans, which has to be considered when using such drugs for chronic studies in dogs. ASMs that are approved for canine epilepsy therapy in Europe are marked by an asterisk while ASMs that are approved for canine epilepsy therapy in the US are marked by two asterisks. In addition, the perceived main mechanism of action of ASMs is shown for each drug. “Mixed” indicates that the ASM acts by more than one mechanism. For details see Rogawski et al. (97) and Sills and Rogawski (98)*.

*BS, binding site; BZD-BS, benzodiazepine binding site; DMD, desmethyldiazepam; MHD, monohydroxy derivative; PAM, positive allosteric modulator; PB, phenobarbital; SV2A, synaptic vesicle glycoprotein 2A*.

a *Active metabolites*.

b *Shortens on continuing exposure to the drug (because of enzyme induction)*.

c *Non-linear kinetics (half-life increases with dose)*.

d *Duration of action independent of half-life because of irreversible inhibition of GABA degradation*.

In dogs that are resistant to the approved drugs, ASMs that are approved for the treatment of human epilepsy can be tried as add-on medication, provided the half-life is long enough to allow maintenance of effective drug levels (cf., Table 1). A variety of such ASMs has been tried as add-on therapy (or monotherapy) in epileptic dogs, mostly with limited success (24, 57, 94). However, levetiracetam has been successfully used for “pulse” treatment for cluster seizures and shortly before generalized convulsive seizures that are predicted by behavioral alterations (93, 99).

Phenobarbital, primidone, and BDZs (e.g., clobazam, clonazepam, and diazepam) lead to tolerance (loss of efficacy) and physical dependency upon chronic treatment of dogs; so the drug dose has to be increased during the 1st weeks of treatment (100). This tolerance is mainly due to the adaptation of the GABA_A_ receptor to the continuous presence of these drugs (functional tolerance); in the case of phenobarbital and primidone, metabolic tolerance (by induction of liver enzymes) contributes to the overall loss of efficacy. With such drugs, abrupt termination of treatment may lead to life-threatening SE. In contrast, imepitoin, which differs from phenobarbital and BDZs, acts only as a partial low-affinity agonist at the GABA_A_ receptor and has no tolerance or dependency liability (101). A further advantage of treatment with imepitoin is that, in contrast to phenobarbital and potassium bromide, no therapeutic drug monitoring (by determination of drug plasma levels) is needed during therapy (101). In this respect, it is interesting to note that therapeutic plasma levels of phenobarbital in epileptic dogs are in the same range (10–40 μg/ml) as those in persons with epilepsy (102).

If ASMs fail to suppress or, at least, ameliorate seizures, there are several additional options to treat the DRE, including the ketogenic diet and vagus nerve stimulation (VNS) (65). However, to my knowledge, only limited proof of evidence is available for such treatments in dogs (see below). In human medicine, precision medicine is being developed for specific types of genetic epilepsies with known etiology (35), but this type of therapy is not available for dogs, yet.

In medicine, many seizure-free patients consider withdrawal of ASMs, both when seizure control is achieved by medication alone, or once they became seizure-free following epilepsy surgery. However, about 30–50% of seizure-free patients who are withdrawn from ASMs will experience seizure recurrence (103). To our knowledge, we were the first to examine how often reinstitution of therapy in people will promptly control epilepsy as before (104). Although seizure control was regained within ~1 year in half of the cases, it took some patients as many as 5–12 years. In addition, in 19% resuming medication did not control epilepsy as before, and chronic DRE with many seizures was seen in up to 23% of patients with a recurrence (104). After our initial report, similar figures have been reported in numerous clinical studies (103). More recently, we examined the same issue in canine epilepsy (105). Following ASM withdrawal, 36% of the epileptic dogs remained seizure-free, but 64% suffered from seizure recurrence, of which only 43% could regain seizure freedom after resuming ASM therapy. Thus, this dog study reflected similar findings in human patients and questioned whether the risk of seizure recurrence is worth the benefit of stopping treatment.

### Randomized Controlled Trials in Epileptic Dogs

Approval of novel ASMs for epilepsy in humans depends on several randomized controlled trials (RCTs), typically performed as add-on therapy in patients with focal epilepsy that is refractory to standard treatments (106). In contrast, in dogs RCTs are also possible in animals with newly diagnosed epilepsy. Furthermore, an added advantage of RCTs in dogs is that US Food and Drug Administration (FDA) agreement is not needed for canine studies unless the drug is being developed for approval in dogs (8). Proof of efficacy by appropriately designed RCTs is available for phenobarbital, potassium bromide, and imepitoin (57). For the latter drug, several RCTs have been performed both in dogs with newly diagnosed epilepsy and in ASM-resistant dogs (57, 101). To our knowledge, we were the first to compare primidone and phenobarbital in a controlled trial in epileptic dogs, showing that phenobarbital is superior to primidone (27), which led to abandoning primidone as a drug of first choice in canine epilepsy. Furthermore, we demonstrated that major ASMs used in humans such as carbamazepine, phenytoin, and valproate are not effective in epileptic dogs because their short half-lives in this species (Table 1) do not allow to maintain effective plasma concentrations during chronic treatment (24).

In contrast, only a few RCTs have been performed in dogs for ASMs that are only approved for human patients (57). As an example, Munana et al. (107) conducted a randomized, placebo-controlled, blinded crossover trial on levetiracetam in dogs resistant to phenobarbital and potassium bromide. Levetiracetam was repeatedly reported to be effective in small non-controlled trials, but in the RCT levetiracetam was not more effective than placebo (107). Nevertheless, as described above, levetiracetam is used as a pulse treatment for seizure prevention in epileptic dogs that are resistant to chronic ASM treatment. For this indication, levetiracetam has the advantage that it is much less sedative than BDZs that are otherwise used for such short and transient pulse treatment. Furthermore, intermittent or pulse treatment with levetiracetam avoids the development of tolerance (loss of efficacy) that has been observed during chronic treatment with this drug in dogs (108) and, initially, in kindled rats (109).

Munana et al. (110) performed similar small RCTs in drug-resistant epileptic dogs with dietary modification and surgical implants, again without any significant difference from placebo. Interestingly, as in humans, a positive response to placebo administration, manifesting as a decrease in seizure frequency, was observed in epileptic dogs (107, 110). This needs to be considered when evaluating open-label studies in dogs that aim to assess the efficacy of ASMs, as the reported results might be overstated (110). There are several explanations for placebo effects on seizure frequency in humans or dogs with epilepsy, including “regression to the mean,” anticipation, classical conditioning, and the natural history of the disease (110, 111). Regression to the mean is a statistical term used to describe the natural fluctuations of seizures that occur over time in a drug trial that typically has a duration of a few months. Epilepsy is a waxing and waning disorder, and fluctuations in seizure frequency are common throughout the disease (112). Dog owners are most likely to seek a change in therapy for their pet (or inclusion of the dog in a drug trial) when seizures are under poor control. Over the short term, improvement in the seizure frequency is probable, regardless of the treatment administered. Thus, drug trials without placebo control may erroneously attribute an improvement in seizure frequency to the drug treatment, whereas in fact, it is because of the effect of time. An alternative to a placebo group is the use of a pseudo-placebo group that is treated with an ASM at a low subtherapeutic dose (113). Furthermore, the superiority of a drug can be demonstrated using a comparative design against a standard ASM.

The latter design was used in a more recent RCT that compared the effectiveness of monotherapy with levetiracetam vs. phenobarbital in dogs with newly diagnosed epilepsy; phenobarbital was effective but levetiracetam was not, even when administered three times daily to take account of the short half-life of this drug in dogs (114). In an RCT to assess the effect of oral cannabidiol administration in addition to conventional ASMs treatment on seizure frequency in dogs with intractable epilepsy, the proportion of responders was similar between the cannabidiol and placebo groups (115). In contrast, a multicenter RCT on a ketogenic medium-chain triglyceride (MCT) enriched diet administered as an add-on dietary supplement had a positive effect on seizure control and behavior in dogs with ASM-resistant epilepsy (116). Furthermore, an RCT on repetitive transcranial magnetic stimulation (rTMS) yielded positive effects on seizure frequency in dogs with DRE (117). Similarly, an RCT on the efficacy of phenobarbital or potassium bromide as add-on ASMs for controlling dogs refractory to a maximum dose of imepitoin resulted in an improvement in seizure management in the majority of the dogs (118).

### Drug Resistance in Epileptic Dogs

DRE occurs when a person has failed to become (and stay) seizure-free with adequate trials of two ASMs (119). Numerous studies suggest that epilepsy fails to be controlled with ASMs in about one-third of adults and ~20–25% of children (28). This condition is also referred to as intractable, medically refractory, or pharmacoresistant epilepsy. Patients with such DRE have increased risks of premature death, injuries, psychosocial dysfunction, and reduced quality of life, so the development of more effective therapies is an urgent clinical need (120). In epileptic dogs, the percentage of drug resistance may even be higher (≥50%) than in humans (121). This may be because the drugs [phenobarbital, primidone (*via* its major active metabolite phenobarbital), imepitoin, potassium bromide] that are approved for the treatment of canine epilepsy all act as positive allosteric modulators (PAMs) at the same target (the GABA_A_ receptor), whereas the many more ASMs approved for humans act by diverse mechanisms (Table 1) (98). Thus, a patient resistant to one mechanistic category of ASMs (e.g., GABA_A_ receptor PAMs) can be switched to another mechanistic category (e.g., ion channel modulators), thereby enhancing the therapeutic armamentarium, whereas this is not possible in epileptic dogs. As in humans, drug resistance continues to be a major clinical problem in the therapeutic management of canine epilepsies with substantial implications for quality of life and survival times (121).

The mechanisms underlying drug resistance in canine epilepsy are only poorly understood. Seizure density and the occurrence of cluster seizures have been linked with a poor response to ASMs (121). Moreover, evidence exists that the genetic background and alterations in epigenetic mechanisms might influence the efficacy of ASMs in dogs with epilepsy (121, 122). Only insufficient data are available in epileptic dogs to support prominent hypotheses of drug resistance in human epilepsy, e.g., the transporter, target, and network hypotheses (120), which will be discussed in more detail below.

Importantly, before defining an epilepsy as drug resistant, pseudo-resistance should be excluded. The main reason for pseudo-resistance in epileptic dogs is poor owner compliance in medical treatment of their pets (123, 124). Another reason may be that the dog is not epileptic but rather has a paroxysmal dyskinesia disorder (125), which, without EEG and knowledge about clinical differences, can be falsely diagnosed as epilepsy (126).

## Pathogenesis of Epilepsy in Dogs

**Figure 2:**
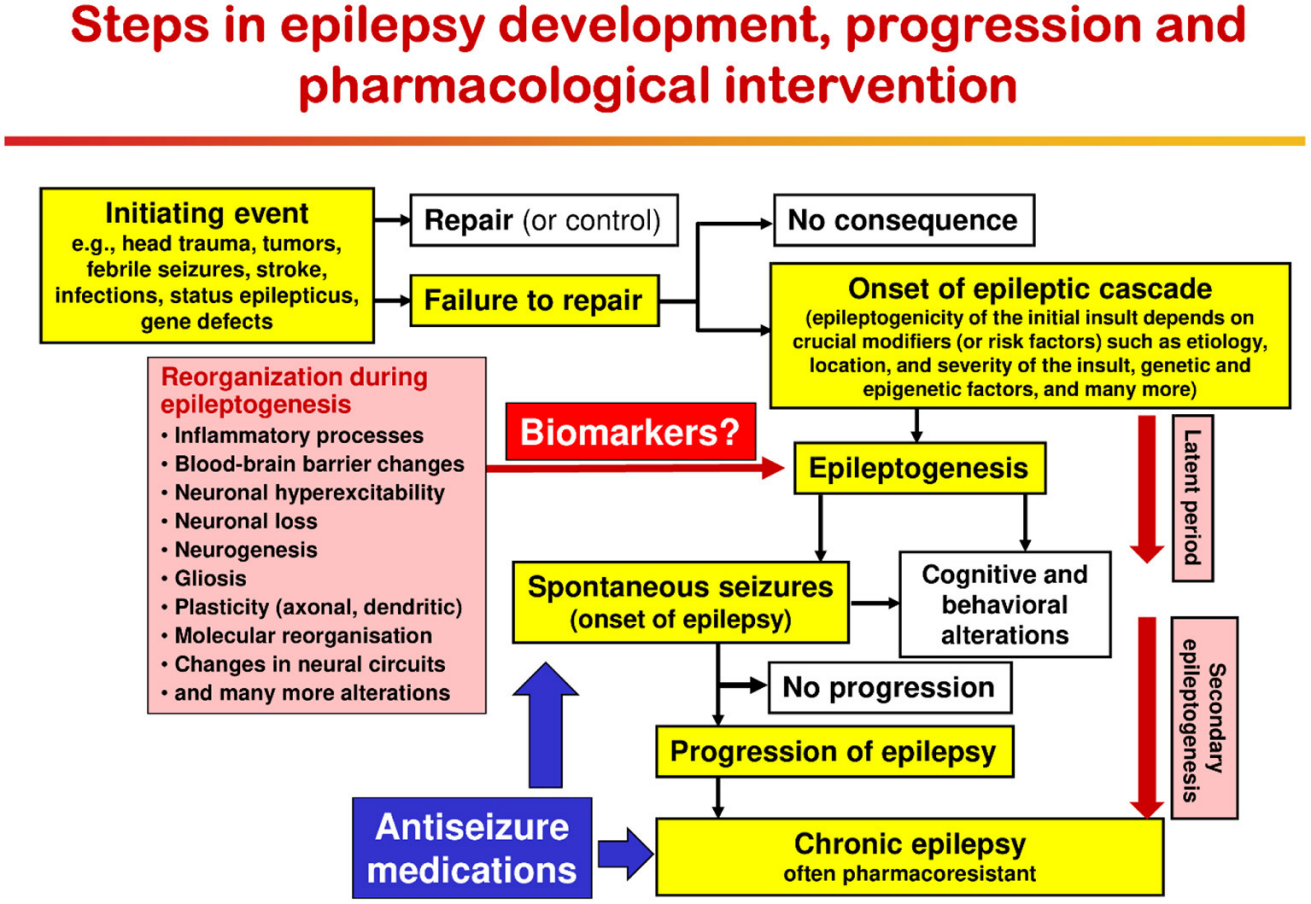
Epileptogenic processes and risk factors involved in the development of epilepsy after acute brain insults. Possibly depending on crucial modifiers or risk factors, the same brain injury can be epileptogenic or not. Immediately after brain injury, early (or provoked) seizures may occur; these acute symptomatic seizures are not indicating epilepsy but may increase the risk of developing epilepsy. In the majority of patients, brain insults do not cause epilepsy. The term epileptogenesis includes processes that render the brain susceptible to spontaneous recurrent seizures and processes that intensify seizures and make them more refractory to therapy (progression or “secondary epileptogenesis”). During epileptogenesis, multiple brain alterations occur, including altered excitability of neurons and/or neuronal circuits, activation of microglia, astrocyte dysfunction, alterations in expression and function of receptors and ion channels (in part recapitulating ontogenesis), loss of neurons, neurogenesis, axonal and dendritic sprouting, gliosis, inflammatory processes, and more. It is important to note that some of these alterations may be related to post-injury repair or recovery and not suited as targets to halt the epileptogenic process. The “latent period” is the time from the initiating epileptogenic brain injury to the first onset of spontaneous clinically obvious seizures. This latent period, during which the epileptogenic processes take place, may last days to months to years. The figure has been modified from previous versions (129–131).

As shown in Figure 1, a variety of brain insults can induce epileptogenesis, i.e., the process underlying the development of epilepsy (Figure 2). In addition, gene mutations underlying inherited epilepsies induce this process. Mainly based on data from rodent models of epilepsy, epileptogenesis is characterized by a variety of structural, molecular, and functional changes in the brain, including inflammatory processes, blood-brain barrier (BBB) disruption, neurodegeneration, synaptic sprouting, plastic changes in ion channels and receptors, and the resultant development of neuronal hyperexcitability in affected brain regions (Figure 2). However, not all patients with the brain insults shown in Figures 1, 2 will develop epilepsy; so biomarkers to predict epilepsy in patients at risk are urgently needed (127). Furthermore, currently, no therapies are available that halt or modify these processes to prevent epilepsy in patients at risk (128). If such therapies would become available, they could also be used to prevent secondary epileptogenesis, i.e., the process leading from newly diagnosed epilepsy to chronic epilepsy, which is often refractory to ASMs (Figure 2).

### Neuroimaging Studies

Except for a few genetic epilepsies, the causes of canine epilepsy are poorly understood. Although the introduction of the MRI as a diagnostic tool of epileptic dogs has disclosed a variety of structural and functional brain abnormalities in such animals (38, 39, 132–136), this by itself does not explain the exact molecular causes of spontaneous recurrent seizures as observed in epilepsy. Furthermore, epileptic dogs, including those with “idiopathic” epilepsy, may have heterogeneous underlying pathologies, including subtle structural changes that cannot be identified on conventional visual inspection of brain MRI.

In a recent peri-ictal MRI study in 81 dogs with suspected idiopathic epilepsy, the most common brain areas affected were the hippocampus (39/81), cingulate gyrus (33/81), and piriform lobes (32/81) (135). This may suggest that, similar to humans, the limbic system (or mesial temporal lobe) is particularly affected in epileptic dogs. This possibility is substantiated by several other MRI studies in large numbers of epileptic dogs (132–134, 136, 137). However, in contrast to human patients, from which epileptic tissue for electrophysiologic and molecular studies can be obtained during epilepsy surgery by resection of epileptogenic focal tissue, such resective surgery is in its infancy in veterinary medicine (66, 138–140).

The use of functional MRI and magnetic resonance spectroscopic imaging (MRSI) in dogs will be discussed in separate sections below.

More recently, positron emission tomography (PET) has been used in epileptic dogs (141, 142). Non-invasive nuclear imaging by PET and single-photon emission computed tomography (SPECT) has significantly contributed to epileptic focus localization in human neurology for several decades (143). Because molecular radiotracer imaging by PET or SPECT offers functional insight into brain alterations, such techniques have the potential for a better understanding of the pathophysiology of epilepsy. Neuro-nuclear imaging in dogs may also serve to identify an epileptic focus in MRI-negative epilepsy. Joint efforts in Finland have led to two recent publications supporting that F-18-fluoro-deoxy-glucose (F-18-FDG) PET for identification of the epileptic focus region as widely used in presurgical evaluation in human patients is translatable to veterinary patients. In juvenile Lagotto Romagnolo dogs with focal-onset epilepsy, Jokinen et al. (141) identified regions with reduced glucose metabolism in the cerebral cortex associated with EEG abnormalities. A second study performed by the same group prospectively evaluated adult Finnish Spitz dogs with focal idiopathic epilepsy by EEG and F-18-FDG and found abnormalities by visual analysis in 9/11 dogs with occipital cortex findings most consistent with the epileptic status (142).

### Brain Tissue Studies

In a postmortem study in an epileptic Shetland Sheedogs, neuronal loss and gliosis were found in the limbic system, including the cingulate gyrus, amygdaloid nucleus, dorsal and ventral parts of the hippocampus, and dorsomedial nucleus of the thalamus (144), which is in line with postmortem findings in human patients with epilepsies originating in the limbic system (145). In a subsequent study in a larger group of epileptic Shetland Sheedogs that died in SE, neurodegeneration and astrocytosis were found predominantly in the cingulate cortex and internal area of the frontal cortex (69). In addition to neurodegeneration, neurogenesis has been reported in the dentate gyrus of an epileptic dog (146), resembling the aberrant neurogenesis in this region reported in humans with temporal lobe epilepsy (TLE) (147, 148). However, in a group of six epileptic dogs of different breeds, which were euthanized because of frequent and severe drug-resistant seizures, no loss of neurons in the dentate hilus and no axonal sprouting were determined, indicating the absence of TLE pathology (149). This is not surprising because only one of the six dogs exhibited focal seizures. Neuron loss in the hippocampus of dogs with epilepsy has been described previously in case reports (144, 150, 151) and a colony of research Beagles (152). Potschka et al. (153) described obvious pathomorphological alterations in canine hippocampal tissue from dogs with both idiopathic as well as symptomatic epilepsy, which would be consistent with data from MRI analyses described above. However, whether TLE exists in dogs remains a matter of debate. Suspected hippocampal sclerosis from MRI scans and volumetry (see above) requires to be substantiated by tissue studies (154).

### Does Mesial Temporal Lobe Epilepsy Exist in Dogs?

In humans, the most common type of epilepsy in adults is mesial TLE (mTLE), an epilepsy syndrome that is characterized by focal (complex partial) seizures originating from the mesial temporal lobe and pathologic lesions, such as hippocampal sclerosis and neurodegeneration in other regions of the temporal lobes (155). For many decades, the limbic system in the temporal lobes, including the hippocampal formation and parahippocampal areas such as the piriform, perirhinal, and entorhinal cortices, have been known to play a crucial role in the development of seizures and epilepsy (156–163). The hippocampus is considered by many to be the generator of mTLE. mTLE is typically associated with hippocampal sclerosis, a neuropathological condition with severe neuronal cell loss and gliosis in the hippocampus, specifically in the CA1 (Cornu Ammonis area 1) region and subiculum of the hippocampus proper and in the hilus of the dentate gyrus (164). In addition to neuron loss, aberrant sprouting of dentate granule cell mossy fibers in mesial TLE is thought to underlie the creation of aberrant circuitry that promotes the generation or spread of spontaneous seizure activity (163, 165). Surgical removal of the sclerotic hippocampus in drug-resistant patients often improves or even cures TLE (120).

The mechanisms by which hippocampal lesions and the associated neuronal network changes within and beyond the hippocampus can lead to enhanced seizure susceptibility and development of recurrent seizures have been the topic of intense research, both in rodent models of mTLE and by using resected tissue from mTLE patients (145). Indeed, in most mTLE patients the seizures originate in this region. However, a very long-standing question and a subject of ongoing debate are whether hippocampal sclerosis plays a role in the development of the epileptic focus or whether it is the consequence of repeated seizures (166, 167).

As discussed above, the relevance of temporal lobe pathology remains a matter of debate in canine epilepsy (54, 56, 153, 154). There have been several reports in the veterinary literature suggesting that mTLE also occurs in dogs. However, in the absence of convincing ictal or interictal EEG abnormalities to confirm that the seizure activity is in the temporal lobe, and with the absence of pathology similar to the human disease (hippocampal sclerosis), there is no definitive evidence that some types of canine epilepsy are actually analogous to TLE in humans. However, several of the MRI and brain tissue data described above strongly indicate an involvement of the hippocampus and other temporal lobe regions in canine epilepsy. Furthermore, many epileptic dogs have a focal seizure presentation that is very similar to that described in humans with mTLE including excessive salivation, staring off, dilated pupils, and facial twitching (32). In line with this, reflecting features of human mTLE, an association between the presence of unilateral epileptic EEG discharges and a decrease in the unilateral hippocampal volume has been described in canine epilepsy (133).

### Brain Microdialysis Studies on GABA and Glutamate

Epilepsy is broadly characterized by aberrant neuronal excitability. Glutamate is the predominant excitatory neurotransmitter in the adult mammalian brain; thus, much of past epilepsy research has attempted to understand the role of glutamate in seizures and epilepsy (168). Glutamate has been implicated in both the initiation and propagation of seizures as well as brain damage that can occur following prolonged or repeated seizures. Gamma-aminobutyric acid (GABA), the most common inhibitory neurotransmitter in the brain, usually suppresses seizure activity. It has long been thought that epilepsy and its increased propensity for recurrent spontaneous seizures are due to an imbalance between glutamatergic excitation and GABAergic inhibition in the brain (169, 170). However, this outdated idea ignores the complexity of the GABAergic and glutamatergic systems in the brain (171). Indeed, experience with GABA indicates that certain neurotransmitters may have either anticonvulsant or proconvulsant effects depending on the neuronal networks, the age, and the pathology involved (172–174).

Despite this complexity of brain neurotransmitter functioning, numerous studies using intracerebral microdialysis of extracellular amino acids in the epileptic focus of human patients undergoing epilepsy surgery have shown marked increases in glutamate release interictally and, more markedly, during seizures (175–180). Extracellular GABA levels were either unchanged or increased during seizures. However, when the release of GABA in the human hippocampus was stimulated by glutamate, it was markedly decreased in epileptogenic hippocampi, in contrast with contralateral, non-epileptogenic hippocampi (181). Intracerebral microdialysis has also been used in epileptic dogs (129). In epileptic Shetland Sheedogs, high values for extracellular glutamate levels were detected in the frontal and parietal lobes in association with an increased number of spikes and sharp waves during hyperventilation. In the cerebrum of Shetland Sheedogs that died of SE, immunohistochemistry using antibodies against glutamate and glutamate transporters (GLT-1 and GLAST) disclosed a decrease of GLT-1 in the cerebral cortex and lateral nucleus of the thalamus (129). These data indicate that the astrocytic uptake of glutamate by GLT-1 is altered in these epileptic dogs, which would explain the increase in extracellular glutamate levels. The GLT-1 findings are of interest because this astrocytic glutamate transporter regulates extracellular glutamate homeostasis in the brain and GLT-1 dysregulation is thought to contribute to the development of epilepsy (182).

### Magnetic Resonance Spectroscopic Imaging Studies on Brain GABA Levels

One inherent problem in measuring extracellular amino acids during epilepsy surgery is the lack of adequate non-epileptic controls. Magnetic resonance spectroscopic imaging (MRSI) can be used to determine GABA in the brain of epilepsy patients vs. controls (183). Indeed, by using MRSI, Petroff et al. (184) reported that persons with mTLE had lower occipital lobe GABA levels than did subjects without epilepsy. MRSI has also been used to study the effect of ASMs that act by potentiating GABAergic transmission on GABA levels, showing that the GABA aminotransferase (GABA-T) inhibitor vigabatrin increases GABA levels in patients with epilepsy (185). In apparent contrast, valproate did not increase significantly GABA concentrations in the occipital lobe of adult patients with complex focal seizures (186). Comparable MRSI studies on brain GABA levels in epileptic dogs are not available but the technique has been evaluated in non-epileptic dogs to measure postictal perturbations of cerebral metabolism following induction of seizures by pentylenetetrazole (PTZ) (187). One disadvantage of measuring brain levels of GABA or glutamate by MRSI is the low spatial resolution of the technique and the fact that only regions such as the occipital lobe can be assessed.

### Studies on CSF GABA and Glutamate Levels

**Figure 3:**
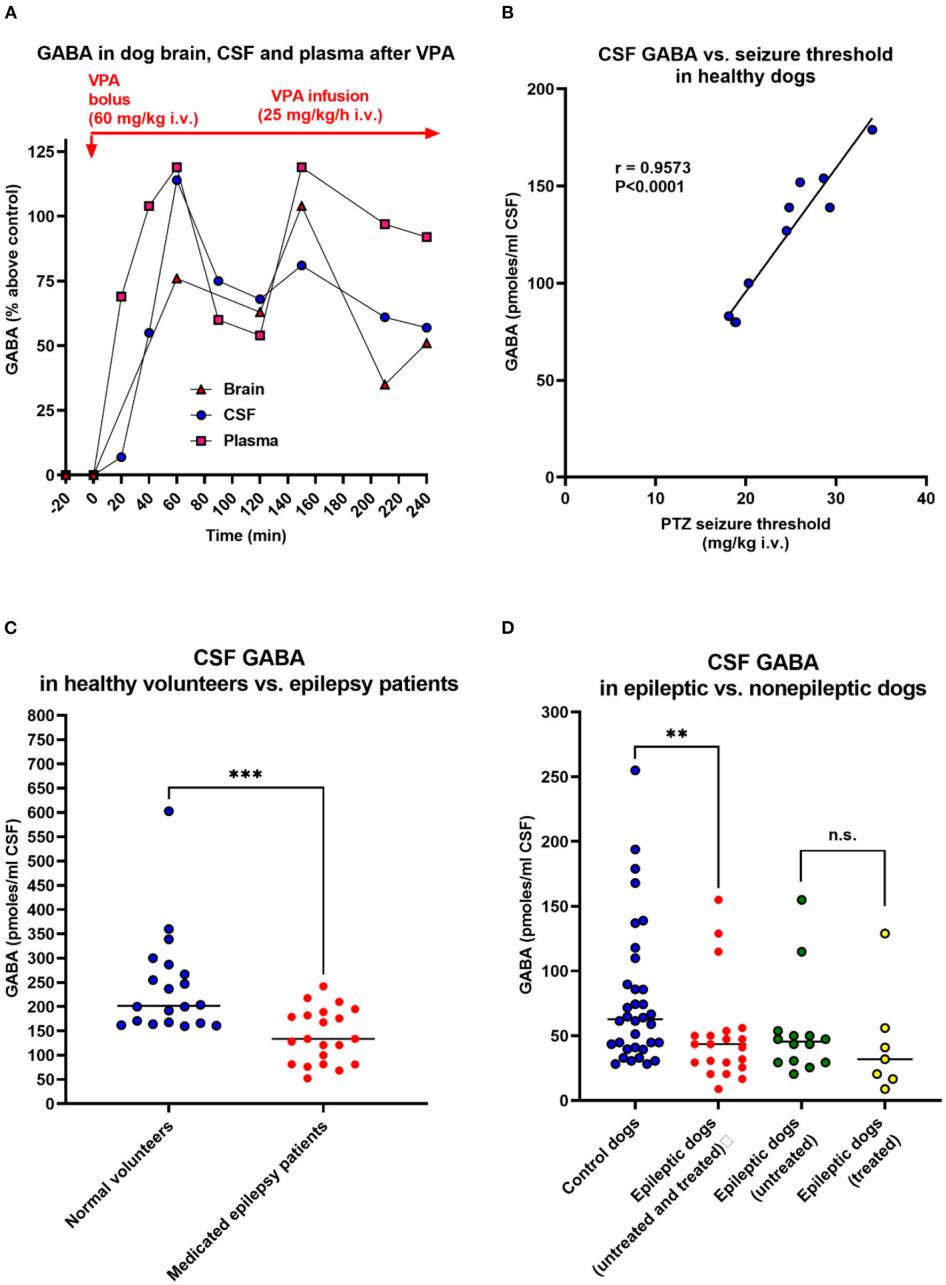
Relationship between CSF GABA concentrations and neuronal excitability. In the dog studies shown in **(A,B,D)**, CSF was withdrawn from the subarachnoidal space by a suboccipital puncture during anesthesia. **(A)** GABA levels in the cerebral cortex, CSF, and plasma during the administration of the antiseizure drug valproate (VPA) in an anesthetized dog. Similar experiments were performed with vigabatrin and other GABA-T inhibitors to investigate the relationship between GABA levels in the brain parenchyma and those in CSF and plasma. Unexpectedly, these experiments showed that plasma GABA alterations reflect respective alterations in the brain and CSF. Also, note the correlation between brain and CSF GABA alterations. VPA is thought to increase GABA synthesis (191), which explains the GABA increases in dogs and other species, including humans (see text). Data are from Löscher (190). **(B)** Correlation between CSF GABA levels and pentylenetetrazole seizure threshold in 10 healthy dogs. Data are from Löscher (192). **(C)** CSF GABA levels in 20 adult unmedicated healthy volunteers and 21 adult epilepsy patients. All patients had more than three seizures a day despite chronic treatment with ASMs (phenytoin, phenobarbital, or primidone). Data are shown as individual lumbar CSF GABA levels and median; the significant inter-group difference is indicated by asterisks (*P* = 0.0003). Data are from Wood et al. (193). **(D)** CSF GABA levels in 34 adult healthy control dogs and 21 adult epileptic dogs. The CSF GABA levels in the epileptic dogs are also shown separately for untreated (*n* = 14) and treated (*n* = 7) dogs, respectively. Data are shown as individual CSF GABA levels and median; the significant inter-group difference is indicated by asterisks (*P* = 0.0075). CSF GABA levels in treated (phenobarbital or primidone) and untreated dogs did not differ significantly. Only one of the seven treated dogs was seizure-free at the time of CSF sampling. Data are from Löscher and Schwartz-Porsche (194).

Another technique to assess extracellular brain levels of amino acids is to determine them in the cerebrospinal fluid (CSF). Close dose-dependent correlations between ventricular or cisternal CSF GABA levels and brain GABA concentrations have been reported following the administration of drugs that elevate brain GABA content (188–190), indicating that CSF GABA levels may reflect brain GABA metabolism and GABA release into the extracellular space. An example in dogs is shown in Figure 3A, in which we compared GABA levels in the brain cortex, CSF, and plasma of an anesthetized dog following administration of valproate, demonstrating impressive parallelism of the GABA alterations. Furthermore, using a seizure threshold model in untreated dogs, we found a highly significant positive correlation between CSF GABA and seizure threshold (Figure 3B), indicating that the concentration of GABA in CSF is related to GABAergic activity in brain compartments involved in the regulation of seizure excitability (192). Moreover, when we kindled dogs by repeated administration of the GABA_A_ receptor antagonist PTZ, the progressive increase in seizure severity was associated with a decrease in CSF GABA levels, which was prevented by the ASM phenobarbital (195). These data thus suggested the usefulness of CSF GABA measurements in clinical investigations of brain GABAergic function. In line with this suggestion, Wood et al. (193) reported that the mean lumbar CSF GABA concentration among 21 medicated epilepsy human patients with intractable seizures was significantly lower than that of 20 unmedicated normal volunteers (Figure 3C). This prompted us to perform a similar study on epileptic dogs (194). As shown in Figure 3D, epileptic dogs exhibited a similar decrease in CSF GABA than previously observed in humans with epilepsy. We also determined CSF GABA in unmedicated epileptic dogs and found no difference to medicated dogs with epilepsy (Figure 3D). Treatment consisted of either primidone or phenobarbital, which are not known to affect brain GABA levels. The similar outcome of CSF GABA studies in epileptic dogs and humans was about the first direct evidence that epileptic dogs may serve as a translational model for the human disease.

In another study in cooperation with pediatric neurologists, we determined CSF GABA levels in children with epilepsy (196–198). Untreated children had significantly lower CSF GABA levels than controls [120 (range 91–159) pmol/ml vs. 174 (range 95–316) pmoles/ml; *P* < 0.02]. The same was true for ASM-treated children with epilepsy when the ASM valproate, which has been reported to increase GABA metabolism (191), was excluded (198). Indeed, valproate was found to increase CSF GABA levels in children with epilepsy by about 100% (197), which is similar to the CSF (and brain) GABA increase with valproate observed in dogs (Figure 3A). A significant decrease in CSF GABA was also found in children with febrile seizures (199).

However, some studies did not report significant decreases in CSF GABA in persons with epilepsy (200–204). At least in part, this could be due to the methods used to determine the low CSF GABA levels, which are known to be sensitive to artifactual increases during sampling, storing, and thawing of CSF samples and GABA analysis (205). More recent studies with modern analytical methods such as electrospray tandem mass spectrometry (ESI-MS/MS) confirmed the initial CSF GABA findings in children and adult persons with epilepsy (206, 207). Similarly, our findings on low CSF GABA in epileptic dogs were confirmed by subsequent studies (208, 209). Interestingly, Podell and Hadjiconstantinou (210) reported that low concentrations of CSF GABA correlate to a reduced response to phenobarbital therapy in epileptic dogs, indicating that low initial CSF GABA is a biomarker of subsequent response to treatment. In humans with epilepsy, the GABA-T inhibitor vigabatrin was found to increase CSF GABA levels (211–214), which was predicted by our studies in dogs (189, 190). Vigabatrin nonresponders had a less marked CSF GABA increase than responders (215). Similarly, treatment of seizures by a ketogenic diet was found to increase CSF GABA in epileptic human patients, with higher GABA levels in responders than non-responders during the diet (216).

In addition to GABA, glutamate levels were measured in the CSF of both dogs and humans with epilepsy. In both species, increases in CSF glutamate concentrations were reported (69, 204, 208, 217–220), although some studies did not confirm these findings (201–203). Such inter-study differences in the outcome of CSF amino acid levels in epilepsy may be due to varying experimental design, patient populations, and ASMs, or the lack of adequate controls.

### Plasma GABA as a Biomarker of Drug Effects

Lumbar puncture for CSF sampling is an invasive method with ethical constraints. Thus, we examined whether drug-induced alterations in the brain and CSF GABA levels are reflected in the plasma. As shown in Figure 3A, surprisingly, the increase in cortical and CSF plasma levels upon treatment of dogs with GABA elevating drugs such as valproate was reflected by plasma GABA levels. Similar findings were reported by us for vigabatrin (189, 190). This prompted us to evaluate plasma GABA as a diagnostic tool for the treatment of human epilepsy patients with valproate and vigabatrin. In both healthy volunteers and epilepsy patients, subchronic treatment with valproate dose-dependently increased plasma GABA levels (221, 222).

Interestingly, a cross-sectional study of epilepsy patients with vigabatrin add-on treatment showed that vigabatrin responders had a significantly higher plasma GABA level than non-responders and controls (223, 224). The possibility of using the plasma GABA increase caused by vigabatrin as a biomarker for the antiseizure response to this drug in patients with drug-resistant focal epilepsy prompted us to perform a prospective clinical study to evaluate changes in plasma GABA concentration in relation to clinical response during vigabatrin treatment of epilepsy (225). Vigabatrin responders had a significant increase in mean plasma GABA both after short-term and long-term treatment, whilst non-responders had no significant changes in GABA levels.

However, while plasma GABA levels parallel drug-induced increases in the brain and CSF GABA, they do not reflect disease-associated alterations in GABA concentrations in the brain (226), although recent studies reported an association between plasma GABA levels and posttraumatic stress disorder symptoms (227, 228). Furthermore, Saleem et al. (229) reported that the plasma levels of GABA and glutamate were significantly higher in patients with DRE compared to healthy controls, but, at least in part, this could be a consequence of the treatment of epilepsy patients with ASMs such as valproate.

To our knowledge, plasma GABA has not yet been evaluated as a potential biomarker in dogs with epilepsy. In our dog experiments with PTZ, plasma GABA levels did not reflect the alterations in CSF GABA in the absence of treatment with ASMs (190, 195). However, as shown in Figure 3A, drug-induced increases in CSF GABA levels of dogs by ASMs such as valproate or vigabatrin were reflected by plasma GABA levels (189, 190, 195). This can be explained by the fact that the GABA degrading enzyme GABA-T is also present in peripheral tissues and blood platelets (230). Furthermore, the GABA synthesizing enzyme glutamate decarboxylase (GAD) is present in some peripheral tissues (230). Thus, drugs such as valproate and vigabatrin that affect GABA-T and/or GAD will increase GABA both in the periphery and the CNS.

### Platelet GABA-T as a Biomarker

Similar to our studies in dogs and humans, experimental studies in rodents have shown that the increase in the brain or CSF GABA concentration induced by vigabatrin or other GABA-T inhibitors, is paralleled by an increase in plasma GABA concentration (188, 231, 232). Interestingly, in children with untreated epilepsy, the activity of GABA-T in platelets was reported to be significantly lower than in healthy controls (233). Surprisingly, patients receiving valproate in monotherapy had a significantly higher GABA-T activity than both the control group and the untreated children with epilepsy (233). As expected, treatment with vigabatrin reduced GABA-T activity in platelets (234). In rats, it was shown that platelet GABA-T reflected the inhibition of GABA-T and increase in GABA levels in the brain after treatment with vigabatrin (235).

Another study in adult patients with focal epilepsy reported that the activity of GABA-T in platelets was increased, but all patients received ASMs such as valproate (236). In apparent contrast to the increased platelet GABA-T, this enzyme was not increased in hippocampal tissue resected during epilepsy surgery. In a study on medicated patients with JME and refractory focal epilepsy, the mean activity of platelet GABA-T in JME patients was significantly higher than in control subjects, whereas focal epilepsy patients did not significantly differ from controls (237). In the latter study, also the GABA uptake into platelets was measured, showing a significant decrease in GABA uptake in both groups of epilepsy patients. Based on the outcome of a study that evaluated GABA and its metabolism and function in platelets as compared to neurons, Kaneez and Saeed (238) proposed that platelets could be further developed to be used as a peripheral model to study neuronal GABAergic function and its abnormality in diseases such as epilepsy.

### The Role of Neuroinflammation

An emerging field in epilepsy research is the assessment of neuroinflammation as a critical process during epileptogenesis as well as in chronic epilepsy (143). PET radioligands of the mitochondrial transmembrane protein TSPO (also known as peripheral BDZ receptor) can be utilized to visualize activated brain resident microglia and brain invading macrophages (239). In addition to patients, TSPO imaging is widely used in animal models of brain diseases (240) but, to our knowledge, not yet in dogs with epilepsy. However, neuroinflammation is routinely being investigated in canine epilepsy by other diagnostic methods (241). Indeed, inflammatory diseases of the CNS are important causes of seizures in dogs (242) and, as in humans, neuroinflammation may be involved in epileptogenesis and ictogenesis, i.e., the processes leading to epilepsy and seizures, respectively. For instance, high-mobility group box 1 (HMGB1), a key mediator of neuroinflammation with increased levels in patients with epilepsy, is significantly increased in the blood serum of epileptic dogs (243). Similarly, dogs with epilepsy had increased levels of interleukin (IL)-1β in serum regardless of the underlying cause of the disease (244). In the CSF of epileptic dogs, significantly higher tumor necrosis factor (TNF)-α and IL-6 concentrations were found (245).

### The Role of the Blood-Brain Barrier

Non-invasive brain imaging methods are also useful to detect alterations in the blood-brain barrier (BBB), which are a hallmark of epilepsy (246). Increased permeability of the BBB leading to extravasation of blood compounds like albumin and subsequent albumin-induced alterations in the brain parenchyma is considered to be a crucial factor for the development of epilepsy (246, 247). *In vivo* imaging approaches to visualize a leaky BBB are based on the detection of contrast agents or radiotracers which do not cross the intact BBB (143). Contrast-enhanced MRI is an established technique to diagnose BBB leakage after epileptogenic insults (248). The latter technique was recently used in 46 epileptic dogs and 6 healthy controls (249). BBB dysfunction (BBBD) was found in 37% of epileptic dogs. The mean BBBD severity score of the piriform lobe in epilepsy dogs was significantly higher compared to control. Furthermore, a significantly higher CSF to serum albumin ratio was found in dogs with BBBD relative to dogs with intact BBB. Brain immunohistochemistry in dogs that were euthanized at the owner's request due to uncontrolled seizures suggested active transforming growth factor (TGF)-β signaling and neuroinflammation in the piriform cortex, showing increased levels of serum albumin colocalized with glial acidic fibrillary protein (GFAP) and phosphorylated Smad 2 (pSMAD2; a downstream signal of activated TGF-β signaling) in an area where BBBD had been detected by MRI. The authors of this landmark study concluded that the involvement of the piriform lobe seen by their MRI protocol emphasizes the possibility of using dogs as a translational model for the human disease (249). One limitation in using imaging methods such as MRSI, PET, or SPECT in dogs is that anesthesia is necessary to achieve immobility of the subject for neuroimaging, which can considerably influence the results of functional brain imaging results (143).

### Genetic Causes of Epilepsies

**Figure 4:**
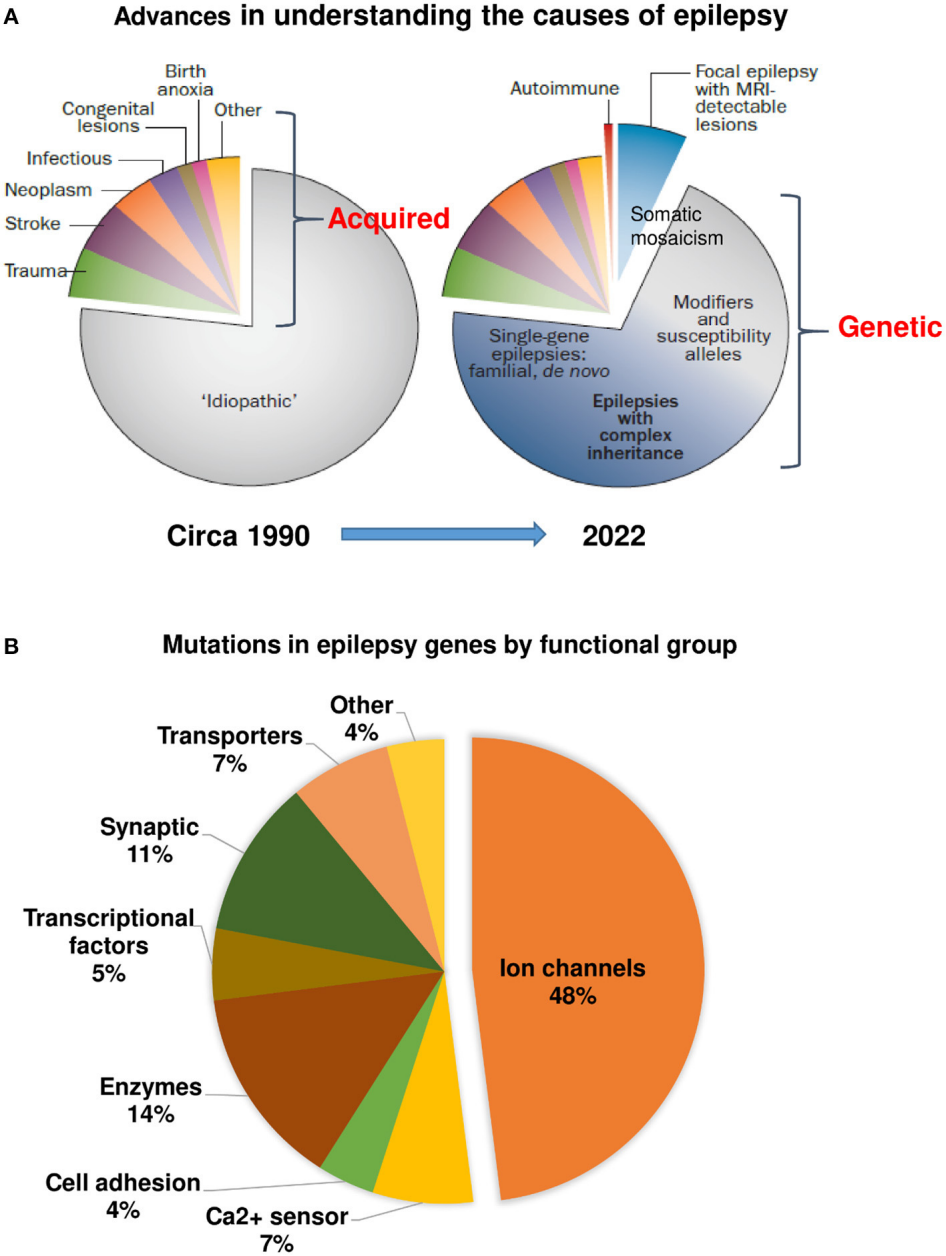
Advances in understanding the causes of human epilepsy. **(A)** Left graph: Till the ~1990's, the majority of epilepsies were characterized as “idiopathic.” Right graph: Today, epilepsy of unknown cause comprises a much smaller proportion, owing to the discovery of autoimmune epilepsies, epilepsies with lesions that are only detectable by MRI, and, most importantly, the reclassification of many epilepsies previously considered idiopathic as having a genetic cause. The exact proportions of monogenic and complex or polygenic epilepsies remain uncertain. Based on Thomas and Berkovic (42) and modified recently by Jeff Noebels. **(B)** Mutations identified in human epilepsy genes by gene function. Modified from Simkin and Kiskinis (37).

Compared to structural, biochemical, and immunological alterations in dogs with epilepsy, much less is known about the genetic causes of epilepsy in dogs. In humans, innovations centered around novel technologies, analytics, and collaboration have led to remarkable progress in gene discovery (45, 250), which has increased our understanding of the causes of epilepsy (Figure 4A). A key difference between the paradigm in the 1990's and today's understanding of epilepsy is that we now have the confidence to leave the term “idiopathic” behind for human epilepsy (42, 250). More than 80 genes are considered as epilepsy genes, i.e., genes that cause epilepsies or syndromes with epilepsy as the core symptom (36). Additional some 800 genes are epilepsy-related or putatively associated with epilepsy. The functions of epilepsy genes are shown in Figure 4B. Most epilepsy genes lead to functional changes in ion channels and cause epileptic channelopathies (37). However, as shown in Figure 4B, various other functional changes, including alterations in receptors for GABA and glutamate, may be caused by epilepsy genes (36). This has resulted in a variety of epilepsy syndromes for which the genetic basis is known (50). The new genomic era now directly affects clinical care toward precision medicine (250). However, monogenetic epilepsies are rare; for most patients, epilepsy is regarded as a complex disorder associated with multiple genes and external environmental factors.

In dogs, many studies of breeds with “idiopathic epilepsy” have failed to identify genes or loci of interest (47, 49, 251). Gene discovery in dogs with progressive myoclonic epilepsies (PMEs) has been more successful, with eight known genes; six of these are orthologous to corresponding human disorders (48, 49). In 2016, Hayward et al. (252) undertook the largest canine genome-wide association study (GWAS) to date, with a panel of over 4,200 dogs genotyped at 180,000 markers, to accelerate mapping efforts. Among these results, the authors found additional candidate genes related to epilepsy in Irish Wolfhounds using 34 cases and 168 controls. In a more recent study that explored the pathogenesis of canine epilepsy using a systems genetics method with Hayward's et al. data (252), combining both GWASs and gene interactions, Cui et al. (253) reported 26 significant subnetworks correlated with canine epilepsy. Combined with gene ontology (GO) enrichment analysis, Cui et al. (253) identified three additional genes that were omitted by the GWAS analysis. Thus, as in medicine, advances in genetic sequencing technologies and bioinformatics are likely to increase the identification of genes and genetic disorders that are associated with epilepsy in dogs. A recent example in this regard is a study on severe early-onset epilepsy in several litters of Parson Russel terriers (254). Combined homozygosity mapping and genome sequencing revealed an in-frame 6-bp deletion in the nuclear-encoded pitrilysin metallopeptidase 1 (*PITRM1*) encoding for a mitochondrial protease involved in mitochondrial targeting sequence processing and degradation. The functional consequences of the mutation were modeled in yeast and showed impaired growth in permissive conditions and an impaired respiration capacity. Postmortem examination revealed an acute diffuse forebrain-predominant necrotizing polioencephalopathy affecting mainly the pyramidal cell layers of the hippocampus proper and entorhinal cortex (254), suggesting an involvement of the limbic system as suggested previously for other types of epilepsy in dogs (see above).

Another interesting recently discovered genetic epilepsy in dogs was described by Wielaender et al. (255, 256). They discovered a novel genetic myoclonic epilepsy in juvenile Rhodesian Ridgeback dogs, characterized by vigorous myoclonic seizures that occur mainly during relaxation periods. More than one-third of affected dogs develop generalized tonic-clonic seizures in the course of the disease and 35% are reported to be photosensitive.

By combining a GWAS and next-generation sequencing analyses using whole-exome and whole-genome resequencing, Wielaender et al. (255) identified a fully penetrant recessive 4-bp deletion in the DIRAS family GTPase 1 (DIRAS1) gene with an altered expression pattern of DIRAS1 protein in the affected brain, including cholinergic forebrain nuclei. However, as in humans, monogenetic epilepsies in dogs are rare, and most idiopathic epilepsies in dogs are either complex disorders associated with multiple genes or are due to as yet overlooked structural alterations as suggested by the data of numerous MRI studies discussed above.

## Spontaneous Recurrent Seizures in Large Inbred Beagle Colonies

Koestner and Rehfeld (257) reported the occurrence of spontaneous generalized convulsions in a large inbred Beagle colony. Redman and Weir (258) found that the incidence of epileptic dogs in a similar colony was 6%. Through selective breeding, this incidence could be increased to 66% (259). A detailed EEG analysis of 10 epileptic Beagles by Wiederholt (260) disclosed limbic hyperactivity in the hippocampus and amygdala, which was associated with psychomotor-like episodes of excessive lipping, smacking, chewing, and drooling, indicating focal-onset seizures. Brain and CSF levels of glutamate did not differ between epileptic Beagles and their non-epileptic siblings (261). In postmortem analyses, neurodegeneration was determined particularly in the hippocampus and cingulate cortex (262).

In addition to idiopathic epilepsy in Beagle dogs, Hegreberg and Padgett (263) described a form of familial epilepsy and its relationship to a similar condition in man, i.e., PME (or Lafora disease), a rare late-onset neurological storage disease characterized by deposits of polyglucosans (Lafora bodies) in the brain and caused by an autosomal recessive genetic defect resulting in myoclonus as well as focal and generalized seizures (264). In addition to Beagles, PME has been described in several other breeds including the Basset hound, Chihuahua, French Bulldog, Pointer, Miniature Poodle, Miniature Dachshund, and Welsh Corgi (49, 265). More recently, PME in Beagle dogs has been characterized in more detail (266).

In a large series of analyses in 68 epileptic Beagle dogs in an epilepsy-prone colony, the most common areas of neuronal damage were the hippocampus, amygdala, piriform cortex, cerebral cortex, basal nuclei, claustrum, septal nuclei, and dorsal thalamic nuclei (152). In addition, intraneuronal inclusions identical to Lafora's bodies were detected in thalamic nuclei of only six dogs (152). Using MRI with voxel-based morphometry, we compared local differences in gray matter volume between 5 healthy Beagles and 10 Beagles with either idiopathic or structural epilepsy (267). Epileptic Beagles displayed statistically significant reduced gray matter volume in the olfactory bulb, cingulate gyrus, hippocampus, and cortex, especially in temporal and occipital lobes.

Epileptic Beagle dogs have only rarely been used for drug testing because the spontaneous recurrent seizures necessitate continuous (24/7) video-EEG monitoring. Instead, as described below, seizures have been induced experimentally in epileptic and non-epileptic Beagle dogs and other dog breeds.

## Experimentally Induced Seizures in Non-Epileptic Dogs

Apart from epilepsy eventually occurring in large colonies of experimentally used Beagle dogs (see above), dogs with epilepsy are typically privately owned pets, which restricts their use as a translational model, because the owners are often not willing to allow invasive experiments or to give away their animals for research purposes (95). In theory, this problem could be resolved by using epileptic dogs from large inbred Beagle colonies (see above); however, the high prime and maintenance costs of dogs in the numbers necessary for selection and breeding of epileptic sublines limit the usefulness of this species for experimental studies (95). Furthermore, the naturally occurring seizures in dogs cannot be elicited at will by an investigator, which makes any scientific studies time-consuming, especially when the seizure frequency is low. However, seizures or SE can be experimentally induced in non-epileptic dogs with many similarities to spontaneous seizures in epileptic dogs. In this regard, we have used PTZ-induced seizures in dogs as a model for drug testing, but seizures can be induced at will by many other convulsive agents in dogs.

As illustrated by the development of imepitoin and also VNS, dogs in which seizures are induced chemically or electrically are a valuable tool both for developing new treatments for canine epilepsy and humans with epilepsy. For canine epilepsy, drug testing in non-epileptic dogs both serves to demonstrate the anti-seizure effect of a new therapy and for dose-finding for first clinical trials in epileptic dogs, which is nicely demonstrated by imepitoin (268). As described in more detail below, following the demonstration of the anti-seizure effect of imepitoin in rodent seizure models, we demonstrated its anti-seizure efficacy in dogs using the PTZ seizure threshold, followed by first clinical trials in dogs with epilepsy (268). The effect on PTZ seizures in non-epileptic dogs correctly predicted its anti-seizure efficacy in epileptic dogs and simplified dose finding in the first clinical trials. This is a huge advantage compared to dose finding when trying to translate preclinical data to first clinical trials in people.

### Seizures Induced by Pentylenetetrazole in Dogs

PTZ, also known as pentetrazol and metrazol, is a CNS stimulant that is widely used experimentally to study seizure phenomena and to identify pharmaceuticals that may alter seizure susceptibility (269). PTZ acts predominantly by antagonizing GABAergic inhibition *via* an effect at the picrotoxin binding site of the chloride ionophore of the GABA_A_ receptor (270). Because of its stimulatory effects on the brain stem, PTZ has clinically been used as a circulatory and respiratory stimulant and, before the invention of electroconvulsive therapy, for convulsive therapy in persons with major depression (269).

**Figure 5:**
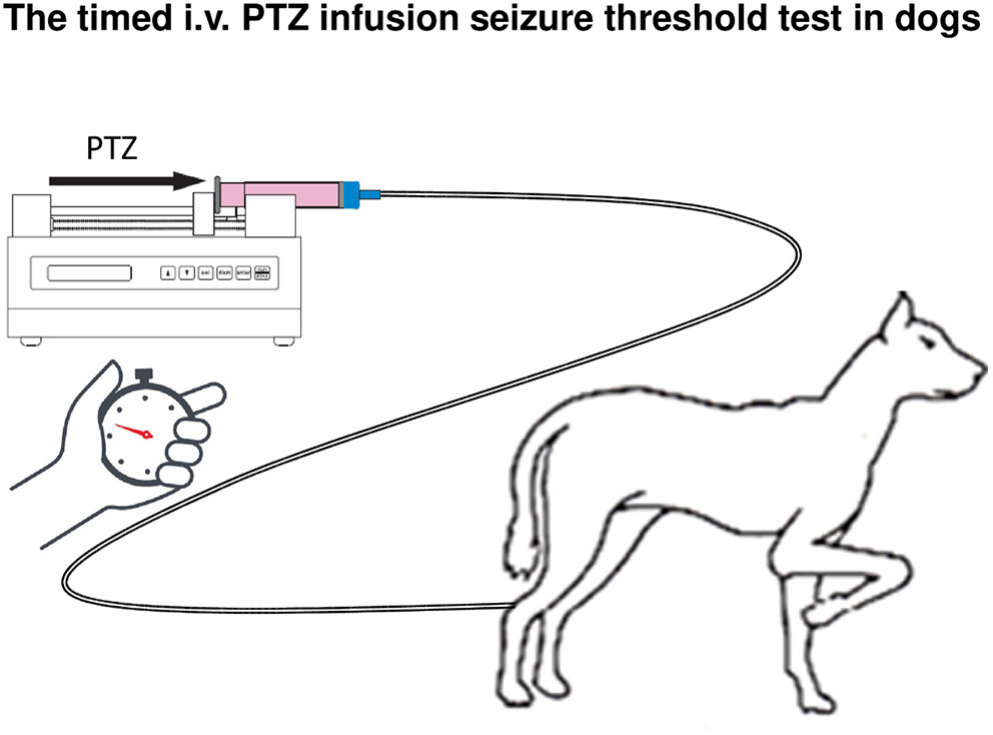
Schematic illustration of the timed i.v. pentylenetetrazol (PTZ) seizure threshold test in an unrestrained dog. Before beginning the experiments, it is important to habituate the dogs to persons involved in the experiments as well as to the rooms and to handling. For seizure threshold determination, a 3% solution of PTZ (in 0.9% NaCl) is continuously infused at a rate of 3 ml/min by an infusion pump *via* a thin, flexible plastic catheter of about 1 m length, connected by a sharp cut-off end of an injection needle to the cephalic vein at a hind leg. The infusion is terminated immediately after the occurrence of the first generalized twitch (initial myoclonus), which usually takes on average 120 s after the onset of PTZ infusion. Before the myoclonic twitch, dogs typically exhibit tremors as a sign of increasing neuronal excitability. During PTZ infusion, the animal is only slightly restricted (or, in trained dogs, not restricted at all). The threshold dose of PTZ (in mg/kg body weight) is calculated from the infusion rate, the bodyweight of the animal, and the time necessary to produce the first myoclonic twitch (which occurs together with the first paroxysmal EEG activity). Typical PTZ seizure thresholds are in the range of ~15 mg/kg PTZ but may vary with the breed, sex, and age of the dogs. The potency of drugs to increase seizure threshold can be determined (and compared) by calculating the doses required to increase the threshold by 20% (TID_20_) or 50% (TID_50_), testing a range of doses in groups of dogs (see **Figure 7**). The same dogs can be repeatedly used at intervals of at least 1 week to avoid kindling (see text). Dogs rapidly adapt to the method and do not show any signs of discomfort or anxiety before or after the threshold determination. Before any drug experiments, the PTZ seizure threshold is determined once per week until reproducible and stable thresholds are obtained in all dogs. The figure was modified from Löscher (269). For details see Löscher et al. (274).

The timed i.v. PTZ infusion seizure threshold test in conscious dogs illustrated in Figure 5 has been developed to test the loss of efficacy (tolerance) developing during prolonged treatment of BDZs such as diazepam (271). In this test, PTZ is infused at a rate of 10 mg/kg per min in 3 ml/min by an infusion pump. The convulsive threshold is defined as the amount of PTZ (in mg/kg body weight) inducing the first generalized myoclonic twitch, at which the infusion is stopped to avoid the development of more severe seizures. In Beagle dogs, the PTZ threshold is typically ~15 mg/kg i.v. At this dose, PTZ induces characteristic paroxysmal discharges in the EEG of dogs (275, 276). An anti-seizure effect is indicated if the PTZ seizure threshold is increased after pretreatment with an ASM or experimental drug, whereas a proconvulsant effect is indicated by a decrease in the PTZ seizure threshold compared to the control threshold. We have used this test in different dog breeds, including Beagles, extensively for determining the development of tolerance to BZDs and related drugs and to testing novel antiseizure compounds (see below). Furthermore, a slow infusion of PTZ is used as a proconvulsant reference compound when employing the dog EEG in safety pharmacology to evaluate proconvulsant risk of test compounds (276, 277). The advantage of using PTZ for induction of seizures is its rapid onset of action, thus allowing to determine seizure threshold during timed i.v. infusion, which is not possible with several other convulsant agents studied in this respect (269, 275).

When the timed i.v. PTZ infusion seizure threshold test is repeatedly used at weekly intervals in the same dogs, no kindling-like increase in seizure response is seen. However, more frequent use leads to a kindling-like effect in that the dogs respond with increasingly more severe seizures to the same dose of PTZ (195).

In addition to PTZ, other GABA_A_ receptor antagonists such as picrotoxin, bicuculline, and penicillin have been used to induce seizures in dogs (275, 278, 279). During i.v. infusion of PTZ or bicuculline in dogs, the most frequent site of the first observed ictal EEG changes was the lateral geniculate body, whereas the neocortex and hippocampus were involved later (279). Furthermore, the glycine receptor antagonist, strychnine, has been used for the induction of seizures in dogs, for instance during the development of VNS (280).

### Electrically Induced Seizures in Dogs

Over several decades, electrical induction of seizures in healthy dogs has been used for different purposes, including neuropathological studies (281), studies on electroconvulsive therapy and depression (282), cardiovascular and neurochemical responses (283), and pharmacological evaluation of experimental drugs (259, 284). For the latter purpose, the maximal electroshock seizure (MES) test, which was developed for ASM screening in mice and rats, was adapted to the dog and pharmacologically characterized by phenobarbital (285). Furthermore, the threshold version of the MES test has been used in dogs and compared with the PTZ seizure threshold (259). However, compared to the timed i.v. PTZ infusion seizure threshold test, the MES test is much less frequently used as a seizure test in dogs, at least in part because the tonic seizures induced by high-current electrical stimulation are much more severe and potentially lethal compared to the short and transient myoclonic seizures induced by PTZ. Interestingly, both the median convulsive current (determined by electric induction) and the PTZ seizure threshold were considerably lower in epileptic vs. non-epileptic Beagles (259). A decreased seizure threshold is thought to be involved in the mechanisms leading to spontaneous recurrent seizures in epilepsy (130).

### Focal Seizures Induced by Topical Penicillin Applied to the Cerebral Cortex in Dogs

In 1945 Walker et al. (286) demonstrated that when penicillin was brought into direct contact with the brain, it produced electroencephalographic and clinical epileptiform manifestations, both in animals and man. The convulsive action of penicillin has since been confirmed in numerous experimental and clinical studies (278, 287–289). Topical penicillin applied to the cerebral cortex produces an epileptic focus in several species, including dogs. The type of focal seizures depends on the exact location of the focus. The convulsive and epileptogenic effects of penicillin result from the blockade of GABA_A_ receptors (289). The penicillin model has been one of the most important models for answering questions about the neuronal basis of epilepsy, including the discovery of the paroxysmal depolarization shift and the analysis of the spread of seizure activity from an epileptogenic focus (288).

In dogs, the penicillin model was used to study the antiseizure effects of magnesium; the effects observed in dogs were then translated to non-human primates (290). Furthermore, the dog penicillin model has been used to study the effects of electrical stimulation of the ninth cranial nerve (the glossopharyngeal nerve) for seizure control in comparison to VNS (291), which will be discussed in more detail below.

### Focal Seizures Induced by Kainate in Dogs

Hasegawa et al. (292) injected the glutamate receptor antagonist, kainate, into the amygdala of Beagle dogs (292). Intra-amygdala or intrahippocampal injection of kainate is a widely used model of TLE in rodents that is characterized by a limbic SE, followed, after a latent period, by frequent spontaneous recurrent seizures that are either focal or generalized convulsive (293). Kainate-treated dogs showed limbic seizures that started from the ipsilateral amygdala and developed into complex focal SE, which lasted for 1–3 days (292). However, in contrast to rodents, the dogs showed no spontaneous seizures during the 2-month observation period. Upon necropsy, severe neuronal loss was observed in the amygdala and hippocampus.

In a subsequent study on this dog model, diffusion-weighted MRI was used to characterize the temporal development of the lesions (294). Furthermore, glutamate and GABA were repeatedly determined in the CSF (295). During the acute phase (3, 6, 12, and 48 h after the onset of SE), CSF-glutamate was significantly increased, while CSF-GABA was decreased, although not significantly. In the chronic phase, both CSF-glutamate and CSF-GABA were significantly lower than normal at 72 h after the onset of SE, and their levels returned to normal at 2 months (295).

### The Kindling Model of Temporal Lobe Epilepsy in Dogs

Kindling is an animal model of TLE produced by focal electrical stimulation of limbic brain areas such as the amygdala, hippocampus, or piriform cortex (296). Furthermore, as discussed for PTZ above, kindling can be induced chemically. The term “kindling” refers to the phenomenon whereby repeated electrical stimulation of a limbic brain region *via* an implanted depth electrode initially only induces focal paroxysmal EEG activity (so-called “afterdischarges”) without overt clinical seizure activity (296). Subsequent stimulations induce the progressive development of focal and later secondary generalized convulsive seizures until the animal is “fully kindled” and responds with the same maximal seizure severity and duration upon stimulation (297). Once developed, the enhanced sensitivity to the initial subconvulsive electrical stimulus is permanent, which is the consequence of enduring molecular and functional brain alterations (296, 298). Since its introduction in 1969 by Goddard et al. (299), kindling has become one of the most widely used animal models of epilepsy, particularly because the mechanisms involved in kindling are thought to be relevant for epileptogenesis (300). Furthermore, the amygdala kindling model of TLE in rats has been instrumental in the preclinical development of various ASMs (301, 302). Electrical kindling has been demonstrated in numerous species, including non-human primates and humans, but is usually being performed in rats (296).

In 1979, Wauquier et al. (303) adapted the amygdala kindling model to Beagle dogs. Kindling in dogs occurred rapidly and did not show the five Racine stages seen in rats. Following once daily stimulation with currents of 50–700 μA, mastication was evoked in the first sessions, but generalized tonic-clonic seizures developed rapidly in all dogs with an intermediate stage of facial clonus, head nodding, and profuse salivation. Fully kindled seizures began with facial clonus, head nodding, and salivation, and were followed by opisthotonos, lifting of the contralateral forepaw, falling over backward, clonicity of the hind legs, tonic extension of the forelegs and hindlegs, quiescence, myoclonic jerking or running seizures; and terminated with wet dog shaking. Following kindling, spontaneous seizures were seen occasionally in all animals, which is an important difference from rodents, which develop spontaneous seizures only after several 100 amygdala stimulations (304). Four of eight kindled dogs died after developing SE. The kindled seizures were completely suppressed by phenobarbital and diazepam, while clonazepam was only partially effective, and phenytoin was ineffective (303).

EEG alterations during amygdala kindling in dogs have been described by Thompson and Galosy (305). Furthermore, kindling has been induced in dogs by repeated electrical stimulation of the olfactory cortex or the anterior piriform cortex (306, 307), which is particularly sensitive to electrical kindling (161, 308).

## The Dog as a Model of Birth Asphyxia and Neonatal Seizures

Birth asphyxia, or impaired gas exchange during the perinatal period, which leads to progressive hypoxia, hypercarbia, and acidosis, is a significant global health problem, responsible for >1 million neonatal deaths each year worldwide (309, 310). Those who survive often suffer from a range of health issues including neonatal seizures and hypoxic-ischemic encephalopathy (HIE). HIE following birth asphyxia is the most common cause of acquired perinatal brain injury and may lead to neurologic sequelae such as epilepsy later in life (311). Neonatal seizures, which typically occur during the first 48 h after birth, are thought to contribute to mortality and morbidity following birth asphyxia (312). Currently, therapeutic hypothermia is the only standard treatment for infants with moderate to severe HIE and it has been shown to reduce both mortality and morbidity (313). However, therapeutic hypothermia has several limitations, so novel therapies for HIE are urgently needed (311). Similarly, currently used therapies (e.g., phenobarbital) for neonatal seizures have limited efficacy (312). Animal models of birth asphyxia, HIE, and neonatal seizures are important to explore cellular and molecular mechanisms, assess the potential of novel therapeutic strategies, and characterize the functional and behavioral correlates of injury (314, 315).

The dog has been used as a “large animal” model for birth asphyxia and neonatal seizures (316–318). For instance, in a series of landmark studies, Duffy et al. have used newborn dogs for studying the effects of asphyxia on cerebral blood flow and metabolism (319–321). Similarly, brain metabolism after induced seizures was investigated in neonatal dogs (316, 322). The “Beagle puppy model of perinatal asphyxia” has been used to study new therapeutic approaches, including calcium channel blockers, glutamate receptor antagonists, non-steroidal anti-inflammatory drugs, and thromboxane synthesis inhibitors (317, 323–327). More recently, neonatal encephalopathy with seizures was described as an autosomal recessive disease of Standard Poodle puppies (328). However, this condition was not associated with birth asphyxia.

## Use of Dogs as a Translational Model in Epilepsy Research and Antiseizure Drug Development

As shown by the review on canine epilepsy above, epilepsy in dogs is similar to human epilepsy in its epidemiology and spontaneity, and its response and resistance to therapy. The similarities make the canine model a promising animal model for testing new therapies, including neurodevices. The advantages of the naturally occurring canine model include that (1) disease surveillance in dogs is second only to that of people; (2) inbreeding in purebred dog breeds makes the genetics easier to determine; (3) drug studies can be done at a lower cost than in people without the need for regulatory approval; (4) full-sized epilepsy monitoring device prototypes can be used in dogs; and (5) dog owners may be willing to try higher risk or unproven therapies if they are on the edge of euthanizing the dog because of poor seizure control (8). The results of canine comparative epilepsy studies are not only of potential translational benefit for people but often also directly help improve the outcome and quality of life for pet's afflicted with epilepsy. The ASM imepitoin, which was initially developed for human epilepsy but—based on dog studies—was approved for the treatment of canine epilepsy, is a good example in this respect (see below).

However, as yet, canine epilepsy is an underutilized model, which has several reasons, including the lack of public awareness about this model in research and therapy development, the marked dog-to-human differences in drug elimination, the less thorough classification of canine epilepsy and epileptic seizures, the lack of routine video-EEG recording, and insufficient knowledge about the etiology of canine epilepsy (8, 61, 153, 329). Nevertheless, as discussed above, there is a relative surge of proof-of-concept studies and RCTs of therapies in naturally occurring epilepsy in the dog in the past ~15 years. These canine studies in the areas of genetics, drug therapy, dietary therapy, implantable EEG devices, and therapeutic devices show proof of concept that canine epilepsy can be a very good model for comparative research for many, but not all, facets of epilepsy, which will be discussed below.

In addition to dogs with naturally occurring epileptic seizures, seizures may be induced by chemical or electrical stimulation in healthy dogs, which increases the applicability of the dog model for the evaluation of new treatments. An important example in this regard is the development of VNS, which was based on a series of studies in dogs with induced seizures (280), which will be described in more detail below.

In the following, I will highlight some of the areas in which the use of dogs, either with naturally occurring or induced seizures, has proved useful in translational research.

### Epileptic Dogs as a Model for Evaluation of Novel Anti-seizure Drugs

**Figure 6:**
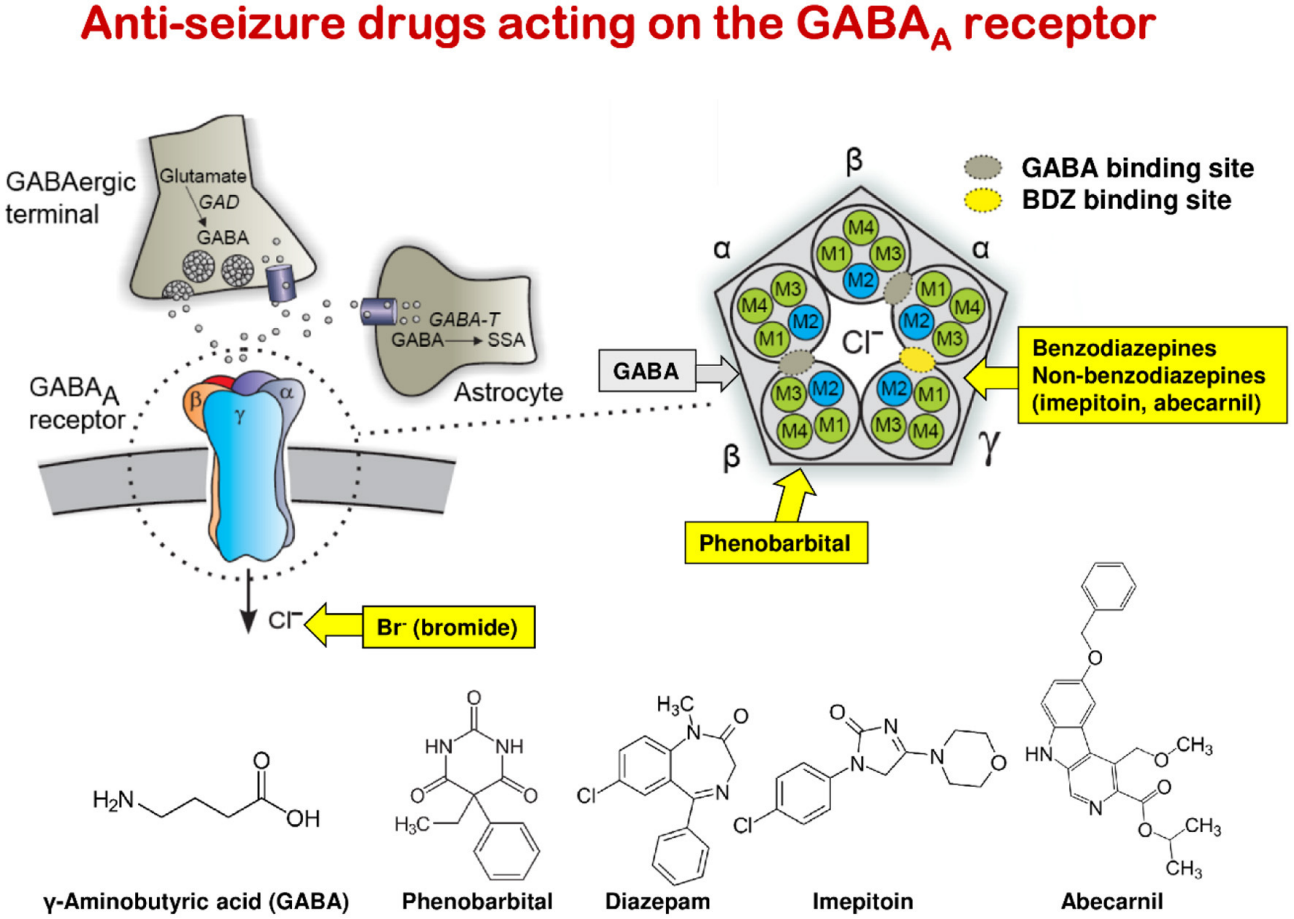
Simplified schemes of the inhibitory GABAergic synapse and the structure of the GABA_A_ receptor illustrating the site of action of benzodiazepines (BZDs) and other drugs acting *via* this site. The left part of the figure illustrates a GABAergic synapse showing synthesis, vesicular packaging, release, uptake, and degradation of GABA in GABAergic nerve terminals; uptake into astrocytes; and the pentameric subunit structure of a typical GABA_A_ receptor complex in the postsynaptic membrane, consisting of α-, β, and γ-subunits. Components of the GABAergic synapse shown include glutamic acid decarboxylase (GAD), the enzyme that catalyzes the decarboxylation of glutamate to GABA; GABA-containing synaptic vesicles (circles containing GABA molecules); the GAT-1 GABA transporter (cylinders); and conversion of GABA to succinic semialdehyde (SSA) by GABA transaminase (GABA-T). The right part of the figure illustrates schematically the pentameric structure of the GABA_A_ receptor within the plane of the neuronal membrane showing the relative positions of the transmembrane domains. Subunit interfaces are formed by M3 and M1. The interfacial locations of the two GABA and one BZD recognitions sites are shown. By binding to the BZD site, BZDs (e.g., diazepam, midazolam, and others), β-carbolines (e.g., abecarnil), and imidazolone derivatives (e.g., imepitoin) act as positive allosteric modulators of GABA leading to increased chloride channel opening frequency, increased chloride influx, and, consequently, to increased hyperpolarization of the membrane and thus inhibition of the postsynaptic neuron. Barbiturates such as phenobarbital also bind to the GABA_A_ receptor to potentiate GABA, but the exact binding site is less well-established. Bromide ions (as produced by administration of potassium bromide) enhance GABAergic inhibition but the mechanism is distinct from that of BDZs and barbiturates in that Br- ions compete with Cl^−^ ions for GABA-gated Cl^−^ channels and, at high concentrations, enter the neuron through these channels, thereby inducing a lasting hyperpolarization of the neuronal membrane. Once Br^−^ ions have entered a neuron, they can only very slowly be eliminated from the neuron, explaining the poor therapeutic ratio and risk of intoxication with potassium bromide. The figure was modified from Rundfeldt and Löscher (101). For comparison, also the chemical structures of GABA, phenobarbital, a BDZ (diazepam), imepitoin, and abecarnil are shown.

Patterson (8) suggested that the canine translational model of epilepsy may well prove to be an excellent intermediate step for confirming preclinical data in rodent models just before initiating human studies. The probably best example in this regard is imepitoin, the first partial “BDZ receptor” agonist developed for the treatment of epilepsy (101). As illustrated in Figure 6, the GABA_A_ receptor exhibits several binding sites, including the GABA recognition site and different recognition sites for BDZs and barbiturates (330). *Via* these sites, BDZs and phenobarbital potentiate the inhibitory action of GABA, thus acting as PAMs. Imepitoin, which is not a BDZ, also acts as a PAM *via* the BDZ site (previously termed “BDZ receptor”), but with much lower affinity and intrinsic efficacy than BDZs (101). Because the anticonvulsant effect of drugs that act *via* the BDZ site occurs at low receptor occupancy, imepitoin exerts potent antiseizure effects in various animal models, but, in contrast to BDZs, does not induce sedation, ataxia, or hypnosis at high doses and lacks tolerance and dependence liability.

**Figure 7:**
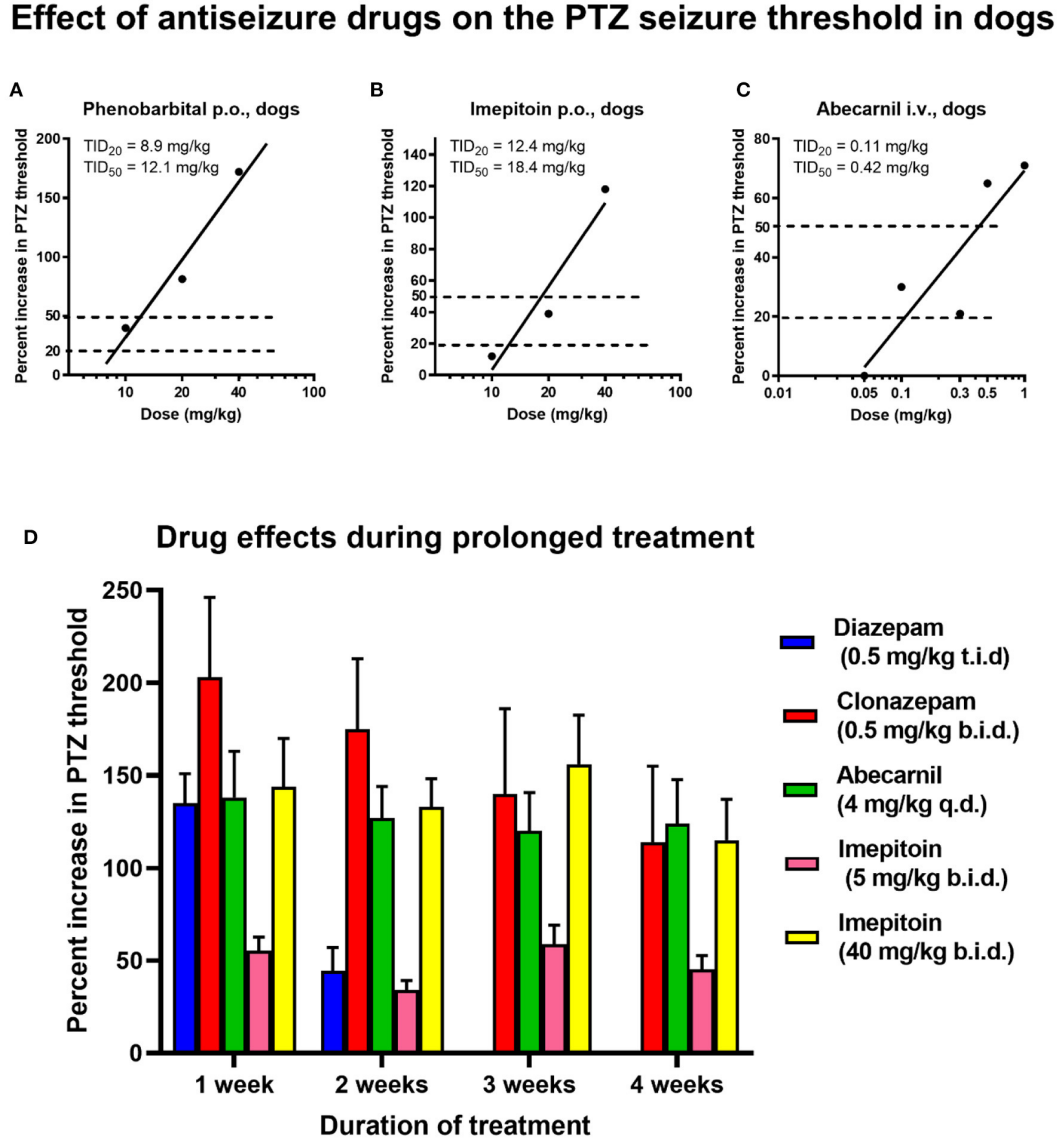
Effect of antiseizure drugs on the timed i.v. pentylenetetrazol (PTZ) seizure threshold test in dogs. **(A)** Effect of different doses of phenobarbital on the PTZ seizure threshold, shown as percent increase above control threshold. Each symbol presents the percent increase in seizure threshold in a group of 6–7 dogs. By nonlinear regression analysis, the doses increasing the seizure threshold by 20% (TID_20_) and 50% (TID_50_) were calculated. **(B)** Effect of different doses of imepitoin on the PTZ seizure threshold. Other details as in **(A)**. **(C)** Effect of different doses of abecarnil on the PTZ seizure threshold. Other details as in **(A)**. **(D)** Alterations in antiseizure efficacy during prolonged treatment in dogs. Four drugs were compared in groups of 4–7 dogs: diazepam, clonazepam, abecarnil, and imepitoin. These drugs were administered daily over 4 weeks. The first PTZ seizure threshold was determined after 1 week of treatment. Control thresholds were repeatedly determined in each dog before the onset of treatment. Data are shown as the drug-induced mean percent increase (± SEM) above control thresholds. Note the rapid decline of antiseizure efficacy of diazepam, indicating the development of tolerance. Tolerance, although less marked, was also observed with clonazepam, but not with imepitoin or abecarnil. Data are from Frey et al. (271), Scherkl et al. (272), Löscher et al. (273), and Löscher et al. (171, 274).

We showed in the timed i.v. PTZ infusion seizure threshold test in dogs that imepitoin dose-dependently increases the PTZ seizure threshold in about the same dose range as phenobarbital (Figures 7A,B), whereas abecarnil, which acts as a high-affinity subtype-selective agonist/partial agonist at the BDZ recognition site, was more potent but not more effective in this model (Figure 7C) (268, 273). Based on the unique pharmacological profile of imepitoin, we also performed chronic studies in dogs and repeatedly determined the anti-seizure effect during treatment (268). Prolonged oral administration with twice-daily dosing of imepitoin with either 5 or 40 mg/kg over 5 weeks was not associated with loss of antiseizure efficacy in the PTZ dog model (Figure 7D). In contrast, as shown in Figure 7D, both diazepam and clonazepam lost efficacy during prolonged treatment in dogs.

The unique properties of imepitoin in dogs prompted us to perform a prospective open-label trial in dogs with newly diagnosed epilepsy (268). The data from the acute and chronic experiments with imepitoin in the PTZ model (Figure 7) were used for choosing doses of imepitoin for the treatment of epileptic dogs. Imepitoin markedly reduced seizure frequency and severity without a significant difference from standard treatments (phenobarbital or primidone) but was much better tolerated than the standard drugs. In dogs with chronic DRE, most dogs exhibited a reduction in seizure frequency and severity during add-on treatment with imepitoin (268). The anti-seizure efficacy and favorable tolerability of imepitoin in epileptic dogs were subsequently confirmed in several RCTs (57, 101) and the drug was approved in Europe for the treatment of canine epilepsy in 2013.

Our dog studies on imepitoin were initially thought to complement the preclinical data before initiating clinical trials in humans. As predicted by the dog experiments, imepitoin proved to be highly tolerable in phase 1 pharmacokinetic and tolerability studies in human volunteers. However, further development for humans was terminated because of pharmacokinetic differences between smokers and non-smokers (101). Instead, it was decided to develop this compound as an ASM for dogs with epilepsy, because two of the scientists (Chris Rundfeldt and Wolfgang Löscher) involved in the development of imepitoin are veterinarians and successfully argued that there is an urgent need for novel ASMs in veterinary medicine.

The β-carboline abecarnil, which as imepitoin did not lose efficacy during prolonged treatment in dogs (Figure 7D), was initially developed as a non-sedative (“anxioselective”) anxiolytic drug for humans (331, 332). However, abecarnil also exerts broad antiseizure efficacies in a wide variety of seizure models, including PTZ seizures in dogs and photically induced seizures in epileptic baboons (273, 274, 333). Abecarnil appears to be a full agonist at α1 and α3 subunit-containing GABA_A_ receptors but is a partial agonist at other receptor isoforms. Abecarnil was not observed to cause significant alterations in motor activity, with the anxiolytic and antiseizure activities of abecarnil typically manifested at doses 3–1,000 times less than those inducing sedation/ataxia and myorelaxation (332). More recently, we evaluated its antiseizure potential in a placebo-controlled pilot study in persons with epilepsy and found marked efficacy (334), which had been correctly predicted by the dog PTZ model (Figures 7C,D).

### Epileptic Dogs as a Model for the Dietary Management of Seizures

Ketogenic diets, which are high in fat and low in carbohydrates, have been used to treat DRE since the 1920's (65, 335). The exact antiseizure mechanism of this diet is not clear, but the ketogenic diet leads to increases in circulating ketones, which may contribute to the efficacy in treating pharmacoresistant seizures (97). Despite a positive effect on seizure frequency when used as an adjunct treatment, most patients discontinue the diet because of its unpalatable and restrictive features. In the last 20 years, new variants of the classical ketogenic diet have emerged (335). Furthermore, the microbiota-gut-brain axis has evolved as a potential target for the ketogenic diet (336). Epileptic dogs have been used both for evaluating novel variants of the ketogenic diet and for studying the potential role of gut microbiota (116, 337–340).

### Epileptic Dogs as a Model for Novel Therapeutic Devices

Concerning devices, the advantage of dogs vs. rodent models of epilepsy is that full-sized epilepsy device prototypes can be evaluated in dogs. As described above, the experimental basis for VNS therapy was a series of studies in the 1980's on the effects of cervical vagal stimulation on seizures induced in dogs (280). In these experiments in mixed breed dogs, seizures or tremors, respectively, were induced by i.v. injection of boluses of strychnine or PTZ at 1- to 4-min intervals until sustained muscle activity was observed electromyographically. Vagal stimulation terminated seizures in 0.5–5 s. Zabara (280) suggested that these results may form the basis of a new therapeutic approach to epilepsy. In line with this suggestion, the results of VNS in dogs were confirmed in a monkey model and shortly thereafter in the first VNS implant in an adult patient with DRE (341). After positive data from two RCTs, the VNS Therapy^®^ System received FDA approval in 1997 for use as adjunctive therapy in reducing the frequency of focal-onset seizures which are refractory to ASMs (341). The first clinical trial using this device in dogs with DRE was published in 2002 (342), reporting up to a 50% reduction in seizure frequency in four of nine dogs. For exploring the neurochemical effects of VNS in dogs, the VNS Therapy^®^ System was implanted in 8 Beagle dogs and levels of serotonin (5HT), norepinephrine, and dopamine were quantified in the CSF after 1 h of sham, standard, and microburst VNS (343). Rapid cycling standard and microburst VNS caused a significant increase of norepinephrine levels in the CSF, whereas no significant changes were detected in 5HT or dopamine levels. These data support previous findings indicating that VNS influences the locus coeruleus-norepinephrine system (344).

In the first long-term evaluation of VNS therapy in a dog with drug-resistant epilepsy, a 5-year-old male Shetland Sheepdog was treated with VNS for 1 year (345). During this period, stimulation parameters were repeatedly adjusted to optimize stimulation intensity while avoiding adverse effects. The frequency of generalized tonic-clonic seizures was reduced by 87% throughout the period of VNS. The owner reported that the dog regained his personality and quality of life.

More recently, Robinson et al. (346) examined the feasibility and efficacy of non-invasive VNS (nVNS) as an adjunct treatment for DRE in dogs. nVNS was found to be safe and easy to administer with mild adverse events. Out of 14 epileptic dogs, nine achieved a reduction in seizure frequency and four were considered responders with a 50% or greater reduction in seizures from baseline to the final treatment period (346).

In addition to VNS, several other types of invasive or non-invasive neurostimulation are available for adjunct therapy for persons with DRE (65). One of the most widely used techniques is RNS, which was approved by the FDA in 2013 (65). Whereas, the total volume of resection surgeries decreased in recent years, RNS implantations have increased by over 100% in persons with DRE (347). RCTs in adult DRE patients have shown that closed-loop RNS to the seizure focus *via* bilateral hippocampal electrodes reduces the frequency of disabling seizures, is well-tolerated, and is acceptably safe (348, 349). The NeuroPace RNS^®^ System, which continuously records the iEEG, recognizes and responds to each patient's unique brain patterns, providing personalized stimulation and preventing seizures before they start. RNS or other types of deep brain stimulation are not yet routinely available for the treatment of DRE in dogs, but dogs played a decisive role in developing the iEEG devices and machine learning algorithms needed to develop closed-loop RNS for human patients. Recently, a novel implantable neural stimulating and recording device was reported to prevent SE events in a dog with severe DRE (350).

Data generated by continuous iEEG monitoring in patients demonstrated that without such monitoring many seizures are missed by the patients or their relatives and caregivers (351). This is certainly similar to seizure documentation such as seizure diaries used by owners of epileptic dogs, which may form a significant bias in trials on new therapies. Indeed, seizure counts based on seizure diaries are often inaccurate and underestimated (351, 352). Similar to the situation in human epilepsy, the unpredictability of seizures plays a major part in the management of canine epilepsy, and dog owners have a strong desire to know when a seizure occurs (353). Automated seizure detection by reliable seizure detection devices would be important to guide treatment decisions or monitor outcomes in clinical trials in both human and canine patients.

### Epileptic Dogs as a Model for Novel Implantable EEG Devices

**Figure 8:**
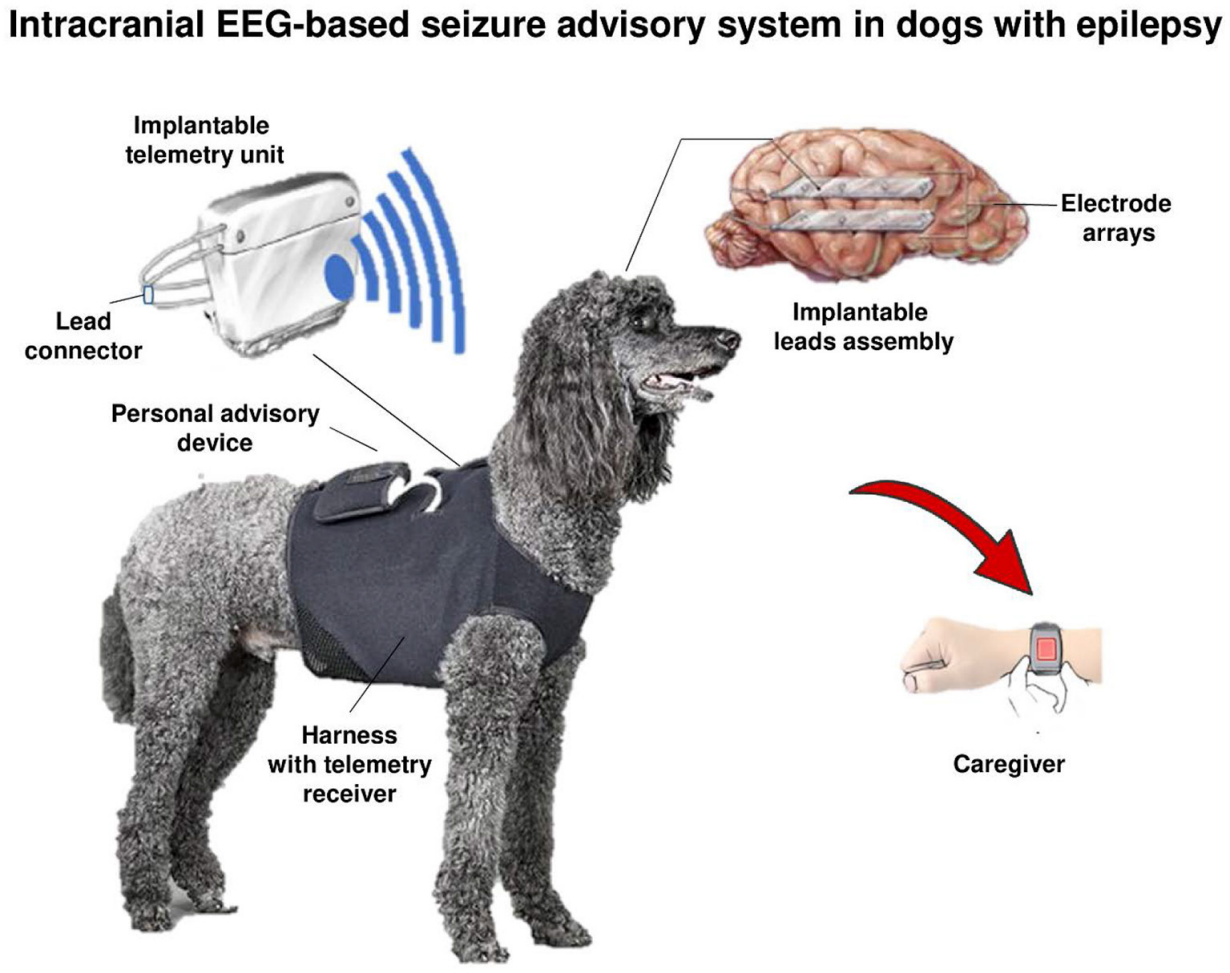
Schematic of a dog with an implanted ambulatory NeuroVista Seizure Advisory System (SAS). The implantable device for recording and storing continuous iEEG includes: An Implantable Lead Assembly (ILA) placed in the subdural space, an Implantable Telemetry Unit (ITU), and a Personal Advisory Device (PAD). The ILA, which acquires 16 channels of iEEG, detects and relays electrical activity in the brain to the ITU. The ITU receives data from the implantable leads, predicts seizure activity using an algorithm, and sends a wireless alert to the PAD. The PAD sends a wireless alert to the caregiver, which may lead to accelerated intervention and administration of seizure-stopping medication (see text). All iEEG data are stored on a flash drive and uploaded weekly *via* the internet to a central data storage site. Modified from Coles et al. (354).

In 2011, Davis et al. (67) described a novel implanted device to wirelessly record and analyze continuous (24/7) iEEG and tested this device in six unsedated epileptic dogs over 5 months (see above). For iEEG monitoring, two electrode arrays with 16 intracranial sensors were placed parallel to the dura in the subdural space to record the iEEG from both hemispheres (Figure 8). The intracranial sensors were coupled to an implanted, rechargeable, subclavicular acquisition and transmission unit, which continuously telemeters iEEG data to an external processing unit for real-time data storage, analysis, and communicating analysis results to caregivers. A seizure detection algorithm, trained on human iEEG data, was deployed on real-time canine iEEG (67).

This device was then used in subsequent studies for mining continuous iEEG in focal canine epilepsy (68, 355). One goal of these studies was to investigate interictal bursts and their electrographic relationship to seizures. Another goal was to focus on the challenges presented by new devices that continuously monitor and process human EEG data over long periods. This work has evolved and improved steadily over recent years, embodied in devices to detect, predict, and respond to seizures in several new implantable devices (348, 349). The large archive of continuous data analyzed for this project (up to 14 months of continuous EEG recording in epileptic dogs) required rigorous, automated methods, including machine learning, for detecting and processing EEG activity. The data obtained further validated canine epilepsy as a promising model of human epilepsy and generated a set of continuous iEEG data of unprecedented length for analysis (68, 355).

Apart from iEEG recording, interesting novel mobile (ambulatory) devices are being developed for continuous scalp or subscalp EEG monitoring in human patients with epilepsy (356, 357). One of these devices uses a subcutaneous (a.k.a. subscalp or subdermal) needle electrode (the UNEEG SubQ™; UNEEG Medical A/S, Lynge, Denmark), which is placed through a minimally invasive surgical procedure under the scalp and contains an inductive coil for transfer of power and data (358). In connection with an external recorder (the 24/7 EEG^TM^ SubQ) combined with a complete data infrastructure and analytics software, the implanted electrode provides continuous (24/7) and automated electrographic seizure detection for up to 15 months (358–360). Several similar subscalp systems have been developed and tested in humans, including the use of such systems for seizure forecasting (359, 361). The size of the subcutaneous needle electrode or subscalp device of the UNEEG system would be ideally suited for continuous EEG recordings in epileptic dogs, which we plan to explore soon. Short-term wireless video-EEG recordings from unsedated epileptic dogs *via* subdermal wire electrodes substantiated the feasibility of such an approach (362).

### Epileptic Dogs as a Model for Seizure Forecasting

Predicting the occurrence of epileptic seizures using machine learning algorithms operating on iEEG or scalp EEG data has the potential to improve the lives of patients living with seizures (363, 364). However, progress toward reliable seizure forecasting has been hampered by a lack of open access to long-duration EEG recordings with an adequate number of seizures for investigators to rigorously compare algorithms and results. In 2014, a large-scale international seizure prediction competition was run on a standard data science contest portal, involving a combination of short-term human iEEG data (with 942 seizures recorded over >500 days) and long-term iEEG data in dogs (348 seizures recorded over 1,500 days) (365, 366). Data from these studies demonstrated the feasibility of seizure forecasting in canine and human epilepsy. Since then, long-duration iEEG recordings from epileptic dogs have been used to further improve the deep-learning algorithms developed for seizure forecasting (367–370). Importantly, the strongest evidence that seizure prediction is possible comes from long-term recordings in epileptic dogs, further substantiating canine epilepsy as a translational model. These studies have shown that seizure prediction performs better than chance in all dogs studied.

### Epileptic Dogs as a Model of Drug-Resistant Seizures

As described above, as in humans, drug resistance is the major clinical problem in the therapeutic management of canine epilepsies. Several RCTs with ASMs in dogs with newly diagnosed epilepsy have shown that at least 50% of the animals do not achieve seizure control (121). Thus, epileptic dogs are an ideal model to study mechanisms of epilepsy (95, 121, 153, 371). To our knowledge, epileptic dogs are the only non-rodent model of epilepsy that allows the selection of animals that respond and do not respond to drug treatment and thus would seem to represent an ultimate tool to study why and how epilepsy becomes intractable. Dogs with ASM-resistant seizures are often euthanized, making postmortem brain studies possible. In this regard, a biobank for canine brain tissue would be useful because this can provide neurologists with a new insight into epileptic brain morphology and identify underlying causes such as cortical dysplasia, which might be overlooked in veterinary medicine and be vital factors for the development of drug-resistance (47).

A detailed examination of dogs with intractable generalized tonic-clonic seizures indicated several differences between resistant and non-resistant animals (95, 121, 372). Dogs with intractable epilepsy have a higher seizure frequency and more severe seizures (including SE and seizure clusters) than epileptic dogs that respond to ASMs, which is consistent with data from prognostic evaluation in humans (121) and in line with the intrinsic severity hypothesis of Rogawski and Johnson (373).

Furthermore, differences in breed distribution were seen in ASM responders and non-responders, indicating that genetics are involved in the medical intractability of canine epilepsy (372). The duration of time for which epilepsy had been present before treatment seemed not to be of major importance for the efficacy of ASM therapy in epileptic dogs.

More recently, the role of the “transporter hypothesis” of DRE has been addressed in epileptic dogs (121). For several major ASMs, drug absorption, distribution, and elimination are affected by drug efflux transporters such as P-glycoprotein (Pgp), which are regulated by promiscuous drug-sensing nuclear receptors and may be overexpressed in patients with epilepsy (120). Efflux pumps such as Pgp are also expressed at the BBB, thus critically reducing functionally relevant drug levels at target sites in the epileptogenic tissue, which may contribute to ASM resistance. Both preclinical and clinical studies have found increased expression of Pgp and other efflux transporters in the area of the epileptic focus of ASM resistant individuals (120, 374). Such increased transporter expression may be a result of frequent seizures or genetic factors or both. For instance, SE in dogs has been reported to increase Pgp in the canine brain (375) and polymorphisms in the Pgp-encoding *ABCB1* gene have been associated with seizure outcomes in Collies with epilepsy (122, 376). Increased BBB expression of Pgp may thus be involved in ASM resistance in dogs, although several other mechanisms certainly contribute to this phenomenon (120). Add-on treatment with the Pgp inhibitor verapamil in dogs with phenobarbital-resistant epilepsy failed to improve seizure control (377). However, verapamil is not a selective Pgp blocker but mainly acts as a blocker of voltage-gated calcium channels, which complicates the interpretation of studies with this drug. Thus, clinical studies with more selective Pgp inhibitors are needed (121). Overexpression of Pgp in the brain and its inhibition can be visualized by PET imaging (378).

Another popular hypothesis of ASM resistance, the “target hypothesis,” suggests that the ASM targets in the brain are altered in DRE (120). Although attractive, the clinical evidence for this mechanism is very limited. Furthermore, the fact that most ASM-resistant patients are resistant to several ASMs acting on different therapeutic targets undermines the general utility of the target hypothesis (374). Recently, it has been suggested that epigenetic mechanisms and protein-protein interactions may result in alterations of diverse drug targets in epileptogenic brain regions, thus explaining why most ASM-resistant patients are resistant to several ASMs acting on different therapeutic targets (121, 379). Seizures and epilepsy induce epigenetic changes in the transcriptome of proteins (including drug targets) by DNA methylation, which may underlie the resistance of several ASMs. However, this interesting hypothesis needs further exploration in both animal models and human and canine epilepsy.

### Status Epilepticus in Dogs as a Model for Novel Treatments

In 2011, Leppik et al. (77) suggested that naturally occurring canine SE may become a translational platform for evaluating the safety and efficacy of interesting compounds with anti-SE activity in rodent studies before their eventual use in human trials. This proposal was based on a randomized, placebo-controlled, double-masked study on i.v. levetiracetam in dogs that presented with convulsive SE to the University of Minnesota Veterinary Medical Center. Dogs that did not become seizure-free with diazepam were enrolled and treated with either levetiracetam or placebo, resulting in a 56% response rate for levetiracetam compared to 10% for placebo (77). Details of the full trial, which also included dogs with acute repetitive seizures (cluster seizures), were published in 2012 (380). Seizure etiologies identified were idiopathic epilepsy (*n* = 10), inflammatory CNS disease (*n* = 4), intracranial neoplasia (*n* = 2), hepatic encephalopathy (*n* = 1), and two dogs had no cause determined. Leppik et al. (77) concluded that—in contrast to rodent models of electrically or chemically induced SE—a test species having naturally occurring SE similar to that in humans and closer in terms of pharmacokinetic characteristics and body size would be very useful. A further advantage is that—in contrast to humans—placebo-controlled studies in dogs are possible because FDA-approved treatments for canine SE are not available. The promising efficacy of levetiracetam in diazepam-refractory (established) canine SE translated to efficacy in established human SE (381).

In 2015, a multi-center proof-of-principle RCT on established canine SE treated with i.v. fosphenytoin, a prodrug of phenytoin, was published (382). Fosphenytoin was significantly more effective than placebo at phenytoin plasma levels within the therapeutic concentration range for people. In a subsequent study, the same group evaluated the pharmacokinetics of i.v. topiramate in dogs with naturally occurring epilepsy (383). Topiramate is not yet approved for i.v. administration, but—based on data from nasogastric tube administration—may be an interesting option for treatment of established SE (384). In this regard, it is interesting to note that we evaluated the safety and pharmacokinetics of a new cyclodextrin-based i.v. formulation of carbamazepine in dogs (385) and demonstrated its efficacy in a mouse model of convulsive SE (386). In 2016, the FDA approved a cyclodextrin-based i.v. formulation of carbamazepine for use in humans.

Recently, Vuu et al. (387) reported the pharmacokinetics, pharmacodynamics, and safety of a new cyclodextrin-based i.v. formulation of allopregnanolone in healthy and epileptic dogs to develop this formulation for early treatment of SE. Allopregnanolone, a neurosteroid that modulates synaptic and extrasynaptic GABA_A_ receptors, was shown to be effective in a mouse model of established SE (388). In the dog study, the rapid onset of effect of allopregnanolone after i.v. infusion suggested that this drug may be useful in the early treatment of SE (387). Overall, these various studies establish the dog as a viable clinical model of human SE.

### Induced Seizures in Dogs as a Model for Novel Treatments

As described above, induced seizures in healthy dogs have been widely used to evaluate new ASMs and therapeutic devices. The most frequently used model has been the timed i.v. PTZ infusion seizure threshold test, which was instrumental in the preclinical development of novel ASMs such as imepitoin. However, the PTZ model is not susceptible to all antiseizure therapies as illustrated by the lack of VNS to increase the PTZ seizure threshold in dogs (343). In this respect, it is important to note that PTZ acts as a GABA_A_ receptor antagonist and, as such, is particularly sensitive to ASMs (such as BDZs, imepitoin, and phenobarbital) that act as PAMs at this receptor. Thus, PTZ cannot replace seizure tests, such as the MES model, which are sensitive to ASMs acting by various other mechanisms. Similar to the MES test in rodents (389), Territo et al. (285) have shown that clonic-tonic seizures in the MES test in dogs can be dose-dependently suppressed by phenobarbital. The authors suggested that MES is a useful model for evaluating generalized convulsions in canines and may provide a tool for a dose selection of novel pharmaceutical compounds before first clinical trials in humans. In a subsequent study, Territo et al. (284) used the MES dog model to further evaluate the antiseizure efficacy of ameltolide, which acts by modulating voltage-dependent sodium channels of presynaptic neurons.

In addition to using the timed i.v. PTZ infusion seizure threshold test for determining ASM efficacies in dogs, this test can be used to determine whether loss of efficacy (tolerance) develops upon chronic administration. Examples are illustrated in Figure 7D. Furthermore, this test can be used to determine whether withdrawal hyperexcitability occurs after rapid termination of treatment. Withdrawal symptoms, including a decreased seizure threshold, spontaneous seizures, or, eventually, SE, are typical for drugs (such as BDZs, barbiturates, and opioids) that induce physical dependence during chronic treatment. For such drugs, withdrawal symptoms do, of course, also occur when no seizures are induced during prolonged treatment, but induction of seizures allows determining tolerance and dependence in the same experiment (100). For drugs that act *via* the BDZ binding site of the GABA_A_ receptor, as an alternative to abrupt termination of treatment, injection of drugs (such as flumazenil) that act as antagonists at this site has been used to precipitate withdrawal symptoms (390). The dog model has been useful to demonstrate that tolerance and dependence can be abolished by developing compounds, such as imepitoin or abecarnil, that act only as partial or subtype-selective agonists at the GABA_A_ receptor (101).

### The Dog as a Model for Pharmacokinetic Studies

As shown in Table 1, most ASMS are eliminated more rapidly by dogs than by humans, which restricts the use of ASMs in canine epilepsy. Despite the dog-to-human differences in elimination kinetics of many ASMs (Table 1), both healthy and epileptic dogs are used to study certain pharmacokinetic aspects of ASMs. For instance, we have used dogs to study the kinetics of CNS entry of several ASMs (16, 18, 23). Similarly, we included dogs when evaluating species differences in the metabolism and plasma protein binding of ASMs (13–15, 391) and characterized the pharmacology of active metabolites, such as the main active metabolite of valproate, in dogs (392). Furthermore, as described above and shown in Table 1, we have determined the pharmacokinetics of various ASMs in dogs as a basis for dose and dosing interval selection for chronic treatment in epileptic dogs (11, 13–17, 19–24, 393). More recently, we studied in dogs whether switching from brand name to generic formulations of phenobarbital is associated with differences in effective drug levels (394).

Numerous other groups have used healthy or epileptic dogs for pharmacokinetic studies, including recent studies on i.v. fosphenytoin (382, 395), topiramate (383), and allopregnanolone (387). Such studies are important to further characterize species differences in drug pharmacokinetics and provide a basis for rational therapy of canine epilepsy or SE. Furthermore, data from invasive studies on drug BBB penetration in dogs can be translated to humans, because BBB characteristics are similar across mammalian species such as dogs and humans (396).

## Conclusion

As shown in this review, because of impressive epidemiological, clinical, and pharmacological similarities, naturally occurring canine epilepsy is an excellent model for human epilepsy. The same is true for canine SE. Even though dogs metabolize most ASMs more rapidly than humans (Table 1), effective plasma levels during chronic epilepsy therapy are remarkably similar, for instance as shown for phenobarbital. Drug resistance is a major problem in both canine and human epilepsy. Similar comorbidities occur in both species. Furthermore, the body sizes of dogs are closer to humans than are rodent body sizes, which makes dose conversion between dogs and humans more accurate. Scaling factors for human equivalent doses are ~1.8 for dogs but ~12 and ~6 for mice and rats, respectively (397). Importantly, dogs are large enough to accommodate therapeutic or iEEG devices designed for humans. Studies in epileptic dogs with such devices have reported ictal events that showed remarkable similarity to human seizures (68, 355). Background EEG and interictal bursts of epileptiform discharges in these animals were also indistinguishable from human iEEG recordings. This work provided a rich dataset of unprecedented length for studying seizure periodicities and developing new methods for seizure forecasting (363).

However, there are also limitations of the epileptic dog as a translational model (329). Despite the efforts of the IVETF to standardize the diagnosis of epilepsy and the assessment of therapeutic responses, the classification of seizures and epilepsy in dogs is controversial. At least in part, this is because the EEG is not a standard procedure in the diagnostic management of canine patients with suspected epilepsy. Furthermore, the use of modern but expensive imaging techniques such as MRI is not a routine procedure in veterinary medicine, although their use is steadily increasing. Concerning studies on drugs or devices in epileptic dogs, such studies are very much affected by the dogs' owner motivation and reliability. Clinical trials in epileptic drugs are as elaborate and time-consuming as trials in human patients, but much less expensive and less limited than studies in humans. Thus, in drug or device development, epileptic dogs should only be used for translational purposes if the additional value is important for the decision of whether to initiate or continue testing in humans. Relevant examples in this regard are studies on novel treatments of SE or novel iEEG devices and seizure forecasting algorithms. Laboratory dogs, such as Beagles, with naturally occurring epilepsy, are an alternative to pets, but the cost and effort to maintain such dogs, along with ethical considerations, pose limitations to experimental epilepsy research in such animals (329).

In summary, dogs with naturally occurring or induced seizures provide excellent large-animal models to bridge the translational gap between rodents and humans in the development of novel therapies. Furthermore, because the dog is not only a preclinical species for human medicine but also a potential patient and pet, research on this species serves both veterinary and human medicine.

## Dedication

This review is dedicated to my late colleagues and friends Drs. Hans-Hasso Frey and Dieter Schmidt who initiated my interest in epileptic dogs as a translational model in the 1970's and performed several studies with me in this species.

## Author Contributions

WL wrote this review and agrees to be accountable for all aspects of the work.

## Funding

The author's own studies in dogs have been funded in part by the Deutsche Forschungsmeinschaft (Bonn, Germany, grant numbers LO 274/xx). This Open Access publication was funded by the Deutsche Forschungsgemeinschaft within the program LE 824/10-1 Open Access Publication Costs and University of Veterinary Medicine Hannover, Foundation.

## Conflict of Interest

The author declares that the research was conducted in the absence of any commercial or financial relationships that could be construed as a potential conflict of interest.

## Publisher's Note

All claims expressed in this article are solely those of the authors and do not necessarily represent those of their affiliated organizations, or those of the publisher, the editors and the reviewers. Any product that may be evaluated in this article, or claim that may be made by its manufacturer, is not guaranteed or endorsed by the publisher.
